# Siglec-F–expressing neutrophils are essential for creating a profibrotic microenvironment in renal fibrosis

**DOI:** 10.1172/JCI156876

**Published:** 2022-06-15

**Authors:** Seungwon Ryu, Jae Woo Shin, Soie Kwon, Jiwon Lee, Yong Chul Kim, Yoe-Sik Bae, Yong-Soo Bae, Dong Ki Kim, Yon Su Kim, Seung Hee Yang, Hye Young Kim

**Affiliations:** 1 Laboratory of Mucosal Immunology, Department of Biomedical Sciences, Seoul National University College of Medicine, Seoul, South Korea.; 2 Department of Internal Medicine and; 3 Biomedical Research Institute, Seoul National University Hospital, Seoul, South Korea.; 4 SRC Center for Immune Research on Non-lymphoid Organs and; 5 Department of Biological Sciences, Sungkyunkwan University, Suwon, South Korea.; 6 Kidney Research Institute,; 7 Department of Internal Medicine, and; 8 Institute of Allergy and Clinical Immunology, Seoul National University Medical Research Center, Seoul National University College of Medicine, Seoul, South Korea.

**Keywords:** Immunology, Nephrology, Chronic kidney disease, Fibrosis, Neutrophils

## Abstract

The roles of neutrophils in renal inflammation are currently unclear. On examining these cells in the unilateral ureteral obstruction murine model of chronic kidney disease, we found that the injured kidney bore a large and rapidly expanding population of neutrophils that expressed the eosinophil marker Siglec-F. We first verified that these cells were neutrophils. Siglec-F^+^ neutrophils were recently detected in several studies in other disease contexts. We then showed that a) these cells were derived from conventional neutrophils in the renal vasculature by TGF-β1 and GM-CSF; b) they differed from their parent cells by more frequent hypersegmentation, higher expression of profibrotic inflammatory cytokines, and notably, expression of collagen 1; and c) their depletion reduced collagen deposition and disease progression, but adoptive transfer increased renal fibrosis. These findings have thus unveiled a subtype of neutrophils that participate in renal fibrosis and a potentially new therapeutic target in chronic kidney disease.

## Graphical abstract

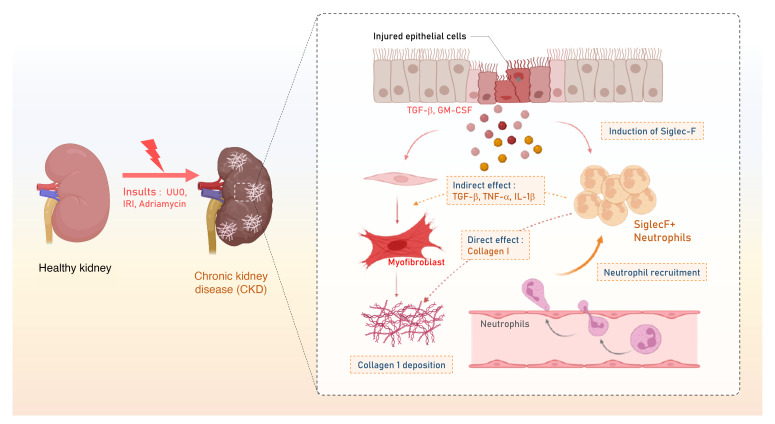

## Introduction

Chronic kidney disease (CKD) is a life-threatening global health problem that is characterized by ongoing and irreversible damage to the structures of the kidney that causes renal function to progressively deteriorate (1, 2). It is generally thought that once CKD starts, it inevitably progresses. The most common cause of CKD is hypertension or diabetes. Therefore, the current treatment strategy for patients with CKD is to delay CKD progression by managing the causative disease (3, 4).

The main pathological features of CKD are progressive nephritis and fibrosis. Renal fibrosis is characterized by the excessive accumulation of extracellular matrix proteins (especially collagen 1), which are largely produced by myofibroblasts (5). Renal fibrosis is triggered and augmented by activated immune cells that infiltrate the kidney and produce profibrotic cytokines (TGF-β1, IL-1, IL-4, and IL-6) and growth factors (PDGF and FGF-2). These immune responses initially aim to resolve the renal injury but ultimately fail to resolve it, resulting in fibrosis (6). Thus, progressive kidney fibrosis could be inhibited by therapeutic strategies that contain, limit, or resolve the excessive inflammation in the kidney.

Several studies have shown that when renal cells die, they release damage-associated molecular patterns (DAMPs), which trigger the infiltration of innate immune cells, particularly neutrophils and macrophages (7–9). While the pathogenic role of neutrophils in CKD remains poorly understood, it is possible that these cells directly injure kidney cells by releasing ROS and granule contents (10). They may also indirectly damage renal cells by producing abundant levels of proinflammatory mediators that attract and activate other immune cells (11). During our preliminary experiments on the roles of neutrophils in CKD, we discovered unexpectedly that kidney damage was associated with large and rapidly increasing numbers of a subset of neutrophils that express the eosinophil marker Siglec-F. This population was discovered only very recently. It was found to respond to DAMP signals and to promote lung tumors, to be present in the infarcted heart, to worsen airway inflammation by air pollutants, and to protect the nasal epithelium from allergy-induced inflammation and bacterial infection (12–19). These studies also showed that compared with conventional neutrophils, the Siglec-F^+^ neutrophils have an enhanced ability to produce ROS and form neutrophil extracellular traps (12, 15, 17–19).

These observations led us to comprehensively study the roles of Siglec-F^+^ neutrophils in the development of renal fibrosis. We showed that Siglec-F^+^ neutrophils became a large immune cell population as fibrosis progressed and that they were generated in the kidney from infiltrating conventional neutrophils by TGF-β1 and GM-CSF. These cytokines are only partly produced by T cells, which indicates that other as-yet-unidentified cells (possibly damaged renal cells and other local immune cells) may also produce these cytokines. We also observed that Siglec-F^+^ neutrophils promoted renal fibrosis not only by producing profibrotic cytokines but also, remarkably, by themselves secreting collagen 1. Importantly, when Siglec-F^+^ neutrophils were transferred after CKD initiation, the progressive fibrosis was markedly increased. These findings together suggest that the Siglec-F^+^ neutrophil/collagen 1 axis in the inflamed kidney may be a potential therapeutic target for CKD.

## Results

### Neutrophils accumulate during unilateral ureteral obstruction–induced renal fibrosis.

**Figure 1:**
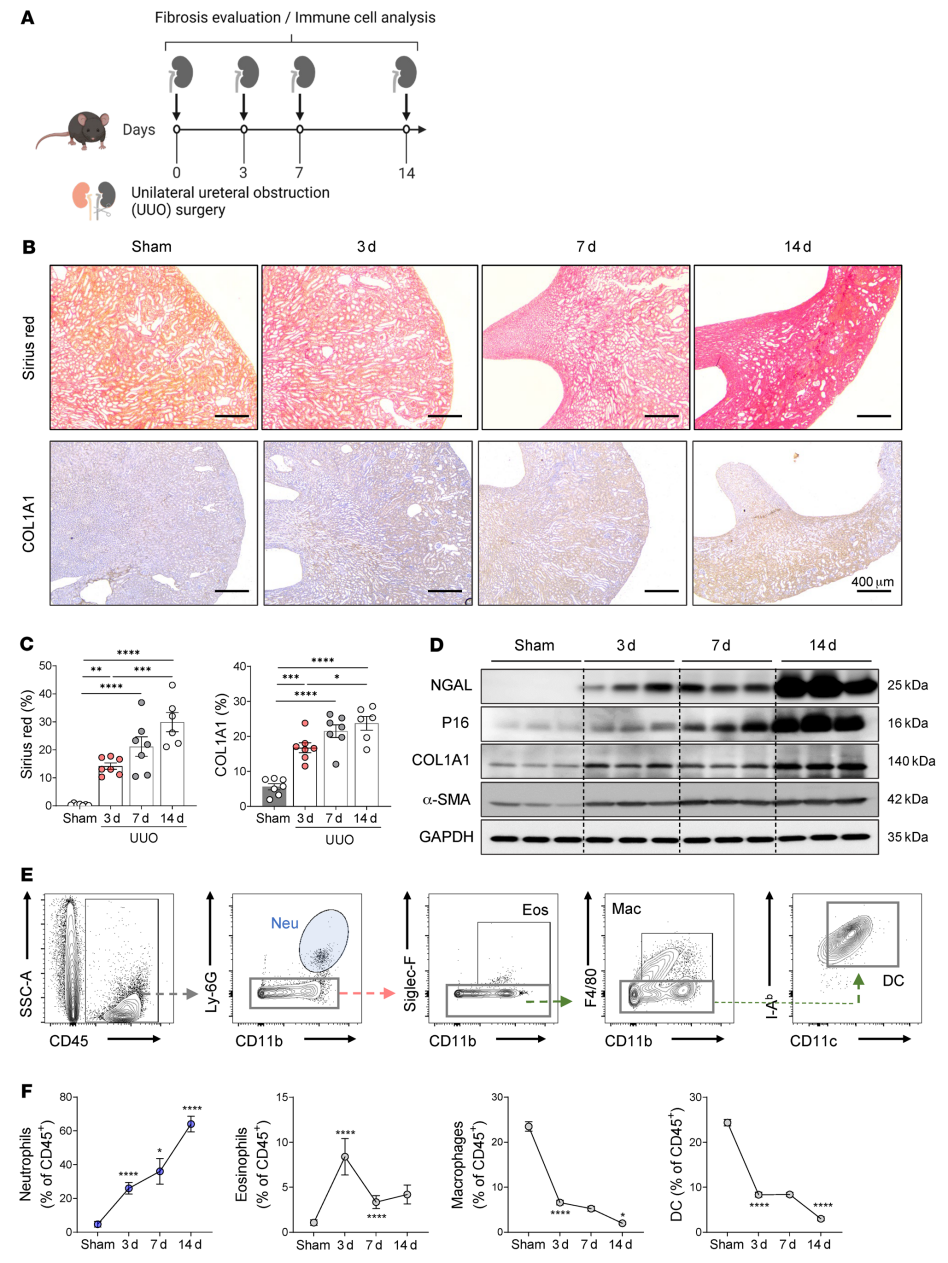
Neutrophils become the most prevalent immune cells in the kidney after fibrosis is induced. (**A**) C57BL/6 mice underwent UUO surgery, after which the fibrosis and immune cell profiles in the kidneys were evaluated on postoperative days 0, 3, 7, and 14. (**B**) Images of Sirius red– and COL1A1-stained kidneys; scale bar: 400 μm. (**C**) Quantification of Sirius red and COL1A1 staining as measures of fibrosis. (**D**) Western blot analysis of the expression of renal injury (NGAL and P16) and fibrosis (α-SMA and COL1A1) markers after UUO. (**E**) Gating strategy used to identify the renal myeloid cells. (**F**) Changes in myeloid cell frequencies during UUO-induced renal fibrosis as determined by flow cytometry. Data are from (**C**) or representative (**F**) of 3 independent experiments. All results are shown as mean ± SEM, and statistical analysis was performed using 1-way ANOVA. *P < 0.05; **P < 0.01; ***P < 0.001; ****P < 0.0001; *n* = 5–7 mice in each group.

The unilateral ureteral obstruction (UUO) model involves the surgical ligation of the left ureter (Figure 1A). It is believed to mimic human chronic obstructive nephropathy and is a widely used animal model for CKD, partly because it rapidly induces kidney fibrosis (20). It caused both tubular cell death and renal fibrosis, as indicated by Sirius red and collagen type 1 alpha 1 (COL1A1) staining. These events, in turn, induced substantial hydronephrosis and loss of renal parenchyma (Figure 1, B and C). These changes were accompanied by Western blot–detected increases in renal injury–related markers over time, including neutrophil gelatinase-associated lipocalin-2 (NGAL) (21), P16 (22), α–smooth muscle actin (α-SMA), and COL1A1 (Figure 1D and Supplemental Figure 1A; supplemental material available online with this article; https://doi.org/10.1172/JCI156876DS1).

When we investigated the immune cell profiles of the UUO-injured kidneys by flow cytometric analysis, we found that the frequency of CD45^+^ leukocytes increased dramatically as renal fibrosis progressed (Supplemental Figure 1B). However, the frequency of lymphocytes (the SSC^lo^FSC^lo^ population) decreased significantly (Supplemental Figure 1C). A closer examination of the CD45^+^ leukocyte subsets then showed that the frequencies of T cells, B cells, NK cells, and type 2 innate lymphoid cells (ILC2s) all dropped after UUO (Supplemental Figure 1, D and E). By contrast, when we looked at the granulocytes, we found that the frequencies of neutrophils (but not eosinophils, macrophages, or DCs) rose significantly as renal fibrosis progressed (Figure 1, E and F). Thus, since the renal immune cell profiling showed that the most prevalent immune cell population in kidneys with advanced fibrosis was neutrophils, this cell type may contribute to UUO pathology.

### Siglec-F–expressing neutrophils accumulate in UUO-injured kidneys.

**Figure 2:**
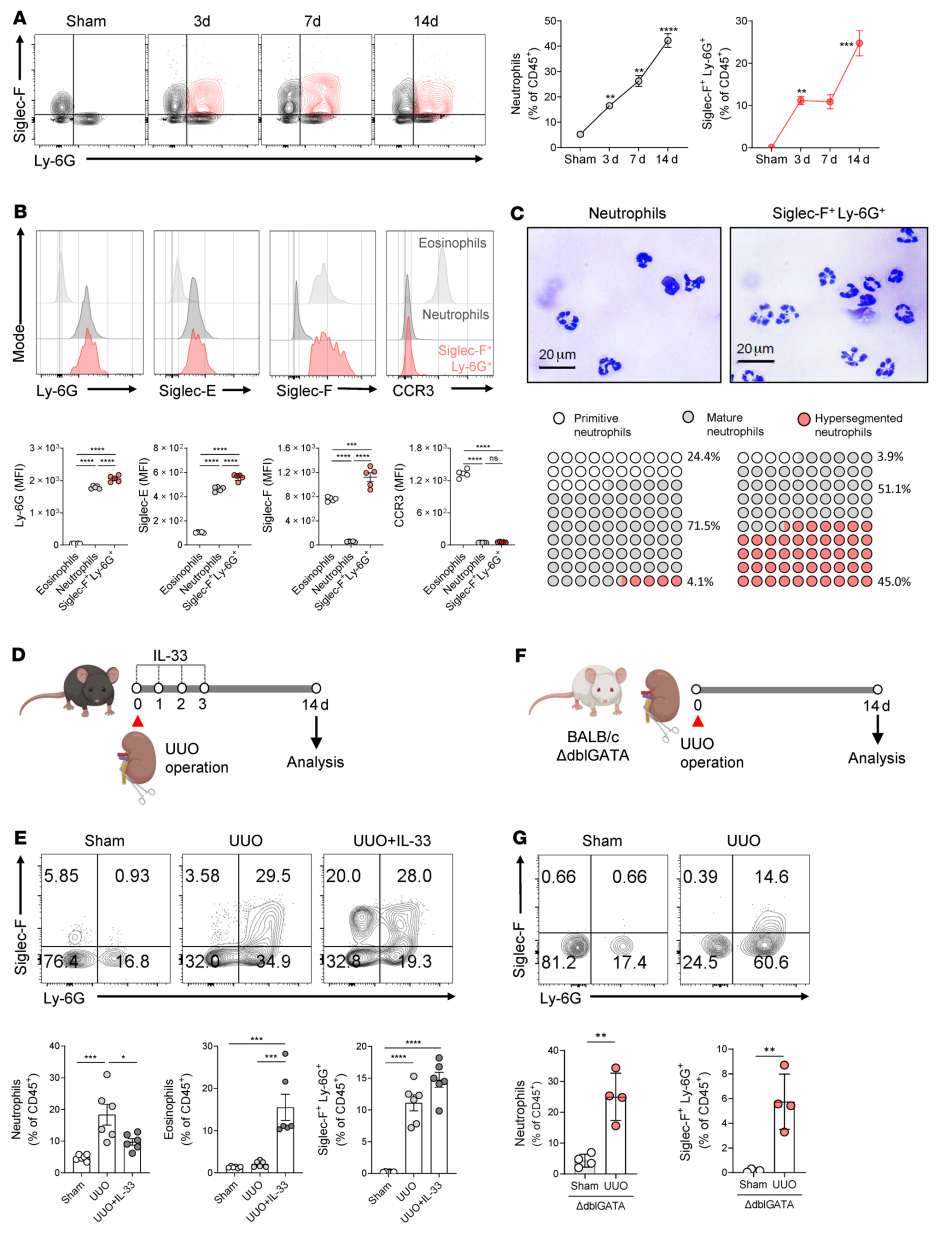
Siglec-F–expressing neutrophils accumulate as fibrosis progresses. (**A**) Flow cytometric analysis of the Siglec-F and Ly-6G expression on CD11b^+^ leukocytes in UUO kidneys. Left, conventional neutrophils (Siglec-F^–^Ly-6G^+^). Right, Siglec-F^+^Ly-6G^+^ cells. (**B**) Flow cytometric analysis of the expression of neutrophil (Ly-6G and Siglec-E) and eosinophil (Siglec-F and CCR3) markers on the conventional eosinophils (Siglec-F^+^Ly-6G^–^), the conventional neutrophils (Siglec-F^–^Ly-6G^+^), and the Siglec-F^+^Ly-6G^+^ cells in the UUO kidney on day 14. (**C**) Evaluation of the morphology of the conventional neutrophils and the Siglec-F^+^Ly-6G^+^ cells on day 14 by sorting and staining them with Diff-Quik and counting the numbers of primitive, mature, and hypersegmented neutrophils; scale bar: 20 μm. (**D** and **E**) Mice were treated with the recombinant IL-33 (250 ng) for 4 consecutive days starting on the day of UUO surgery (**D**), and the frequencies of conventional neutrophils, eosinophils, and Siglec-F^+^Ly-6G^+^ cells on day 14 were determined by flow cytometry (**E**). (**F** and **G**) Eosinophil-deficient ΔdblGATA mice (BALB/c background) were subjected to UUO (**F**), and the conventional neutrophil and Siglec-F^+^Ly-6G^+^ cell frequencies in the kidney on day 14 were determined by flow cytometry (**G**). All results are shown as mean ± SEM, and statistical analysis was performed using 1-way ANOVA (**A**, **B**, and **E**) or Mann-Whitney *U* test (**G**). *P < 0.05; **P < 0.01; ***P < 0.001; ****P < 0.0001; *n* = 4–5 mice in each group.

When we analyzed the surface markers of the neutrophils in the fibrotic kidneys further, we unexpectedly found an Ly-6G^+^ neutrophil population that coexpressed the eosinophil-specific surface marker called Siglec-F (Figure 2A). A kinetics analysis then showed that these Siglec-F^+^Ly-6G^+^ cells were extremely rare in the kidney at baseline but that as inflammation and fibrosis progressed in the UUO-injured kidney, their frequency rose to 24% of the CD45^+^ leukocytes at 14 days (Figure 2A and Supplemental Figure 2A). The conventional neutrophils (Siglec-F^–^Ly-6G^+^) also increased after UUO from 5% of the CD45^+^ leukocytes at baseline to 42% at 14 days (Figure 2A). This change was observed regardless of whether UUO was conducted in C57BL/6 (Figure 2A) or BALB/c mice (Supplemental Figure 2B).

Our analysis of the phenotypic characteristics of these populations then showed that the Siglec-F^+^Ly-6G^+^ cells and the conventional neutrophils both expressed the neutrophil marker Siglec-E but not the eosinophil marker CCR3, whereas the eosinophils expressed Siglec-F and CCR3 but not Siglec-E or Ly-6G (Figure 2B). Moreover, our morphological analyses showed that the Siglec-F^+^Ly-6G^+^ cells showed multilobulated features like conventional neutrophils. Interestingly, this population had a higher frequency of hypersegmented cells and was more likely to display higher forward scatter and side scatter than the conventional neutrophils (Figure 2C and Supplemental Figure 2, C and D). Notably, Ogawa et al. reported that Siglec-F^+^ neutrophils in the inflamed olfactory neuroepithelium coexpress the macrophage marker F4/80 (17). However, Siglec-F^+^Ly-6G^+^ cells in the kidney did not express the macrophage marker F4/80, confirming they were not contaminated with renal macrophages (Supplemental Figure 2E).

To confirm that the Siglec-F–expressing neutrophils were truly distinct from eosinophils, we injected mice with recombinant IL-33 on days 0, 1, 2, and 3 after UUO (Figure 2D). IL-33 regulates the expansion of eosinophils (23): if this population is an eosinophil population, this treatment will expand them in the kidney. However, while IL-33 treatment significantly increased the eosinophil frequencies, it did not significantly increase the frequencies of the Siglec-F^+^Ly-6G^+^ population (Figure 2E). Moreover, the Siglec-F^+^Ly-6G^+^ population could be generated by UUO in eosinophil-deficient ΔdblGATA mice: on day 14, these mice and the control BALB/c mice had similar frequencies of Siglec-F^+^Ly-6G^+^ cells (6% vs. 4%) (and conventional neutrophils, 25% vs. 23%) (Figure 2, F and G, and Supplemental Figure 2B). Together, these results showed that Siglec-F^+^Ly-6G^+^ cells are indeed neutrophils that are distinct from conventional neutrophils in their Siglec-F expression and higher levels of hypersegmentation, larger size, and greater granularity. Henceforth, we will refer to this population as Siglec-F^+^ neutrophils.

### UUO-induced Siglec-F^+^ neutrophils are localized in the damaged kidney and arise from conventional neutrophils in the renal vasculature.

**Figure 3:**
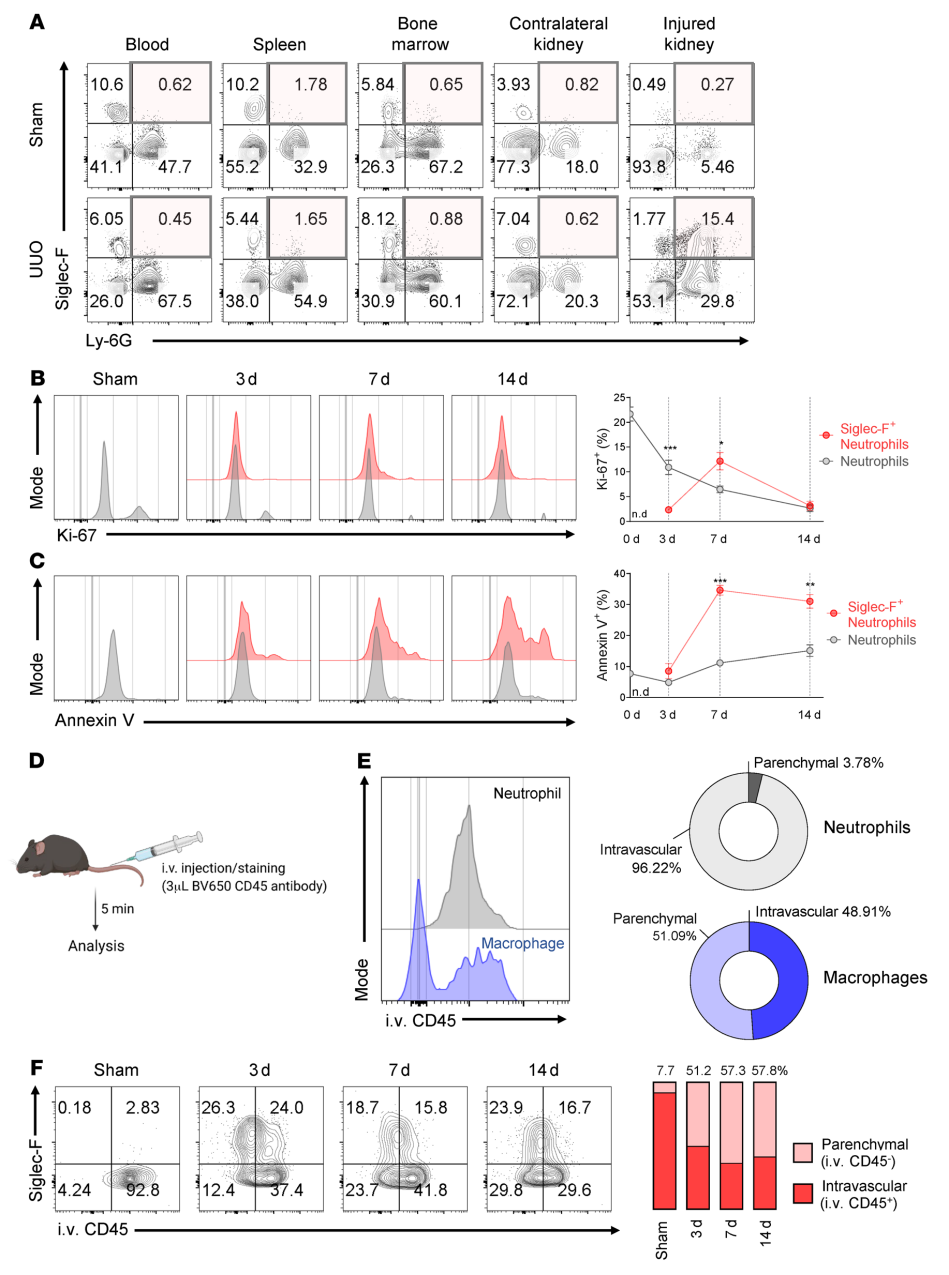
UUO-induced Siglec-F^+^ neutrophils arise only in the injured kidney and are derived from conventional neutrophils in the renal vasculature. (**A**) Flow cytometric analysis of the frequencies of Siglec-F^+^ neutrophils in other organs (blood, spleen, bone marrow, and the contralateral kidney) as well as the injured kidney in UUO-treated mice on day 7. (**B** and **C**) Kinetic changes of the Siglec-F^+^ and conventional neutrophil frequencies in terms of cell proliferation (Ki-67 staining) (**B**) and cell death (annexin V staining) (**C**). (**D**–**F**) Sham- and UUO-treated mice were i.v. injected with a BV650-labeled CD45 mAb 5 minutes before they were euthanized (**D**). As a control to confirm that the mAb labeled the leukocytes in the kidney vasculature but not in the kidney parenchyma, naive mice were injected with the BV650-CD45 mAb and the neutrophils and macrophages in the kidneys were subjected to flow cytometry (**E**). The BV650-CD45–labeled (intravascular) and BV650-CD45–unlabeled (parenchymal) Siglec-F^+^ neutrophil frequencies in the UUO-damaged kidney over time were determined by flow cytometric analysis (**F**). All results are shown as mean ± SEM, and statistical analysis was performed using Mann-Whitney *U* test (**B** and **C**). *P < 0.05; **P < 0.01; ***P < 0.001; *n* = 4–5 mice in each group. n.d., not detected.

To elucidate the origin of the Siglec-F^+^ neutrophils in the fibrotic kidney, we searched for this population in the peripheral blood, spleen, bone marrow, and contralateral kidney in the sham- and UUO-treated mice. Siglec-F^+^ neutrophils were barely present in peripheral blood, spleen, and bone marrow in both the sham- and UUO-treated mice. However, Siglec-F^+^ neutrophils accounted for 15.4% of the CD11b^+^ myeloid cells in the damaged left kidney (0.27% in the left kidney of the sham-treated mice). By contrast, the conventional neutrophils increased in the peripheral blood and spleen in the UUO-treated mice (Figure 3A). The latter observation is consistent with the current understanding of CKD as a systemic disease (24). Therefore, we hypothesized that Siglec-F^+^ neutrophils were generated from the conventional neutrophils in the inflamed renal lesions rather than originating from outside the injured kidney.

To test this, we first asked about the proliferative activity of Siglec-F^+^ neutrophils and conventional neutrophils. Ki-67 staining of these populations in the injured kidney showed that although 22% of the conventional neutrophils were proliferating at baseline, this dropped rapidly over time to low levels (3%) on day 14. The Siglec-F^+^ neutrophils showed a very mild rise in proliferative activity on day 7, after which it dropped to low levels on day 14. Thus, neither population demonstrated any marked proliferation (Figure 3B). We also noted that while the conventional neutrophils showed low but rising apoptotic activity over time, the Siglec-F^+^ neutrophils rapidly became highly apoptotic (Figure 3C). These results suggest the increase in the Siglec-F^+^ neutrophil population in the damaged kidney (as shown in Figure 2A) was due to continuous replenishment of these cells, but that this replenishment did not come from proliferating Siglec-F^+^ neutrophils; instead, it appears that the Siglec-F^+^ neutrophils were generated from the conventional neutrophils.

We next asked, where are the conventional neutrophils converting to Siglec-F^+^ neutrophils? We knew the peripheral blood was not the location because Siglec-F^+^ neutrophils were not found in this specimen (Figure 3A). Thus, the conversions were occurring either in the parenchyma of the damaged kidney or in the renal blood vessels. To determine which one, UUO-treated mice were i.v. injected with a BV650-labeled CD45 mAb 5 minutes before the animals were euthanized, and the intravenous (i.v.) CD45^+^ cell populations in the kidney were analyzed (Figure 3D). This injection yielded 2 lymphocyte populations, namely, the BV650-CD45^+^ leukocytes in the renal blood vessels and the BV650-CD45^–^ leukocytes in the kidney parenchyma. As a control for this experiment, we examined the renal parenchymal and vessel locations of neutrophils and macrophages of CD45 mAb-injected naive mice. As expected, nearly all of the neutrophils in the kidney of the naive mice were circulating cells in the renal blood vessels, whereas half of the macrophages were in the kidney parenchyma and half in the blood vessels (Figure 3E). When we examined the Siglec-F^+^ neutrophils in the UUO-injured kidney on days 0, 3, 7, and 14 after the BV650-CD45 mAb injection, we found that the few Siglec-F^+^ neutrophils were present on day 0 and nearly all in the renal vessels. By day 3 after UUO, half of the much more abundant Siglec-F^+^ neutrophils were in the parenchyma, with the other half being located in the renal blood vessels, which continued to be observed on days 7 and 14 (Figure 3F). This supports the notion that UUO causes conventional neutrophils in the renal blood vessels to convert to Siglec-F^+^ neutrophils, which then migrate into the renal parenchyma. Since these conversion events are ongoing, they both replace the apoptotic Siglec-F^+^ neutrophils and further expand this population over time.

### TGF-β1 and GM-CSF induce Siglec-F expression in neutrophils.

**Figure 4:**
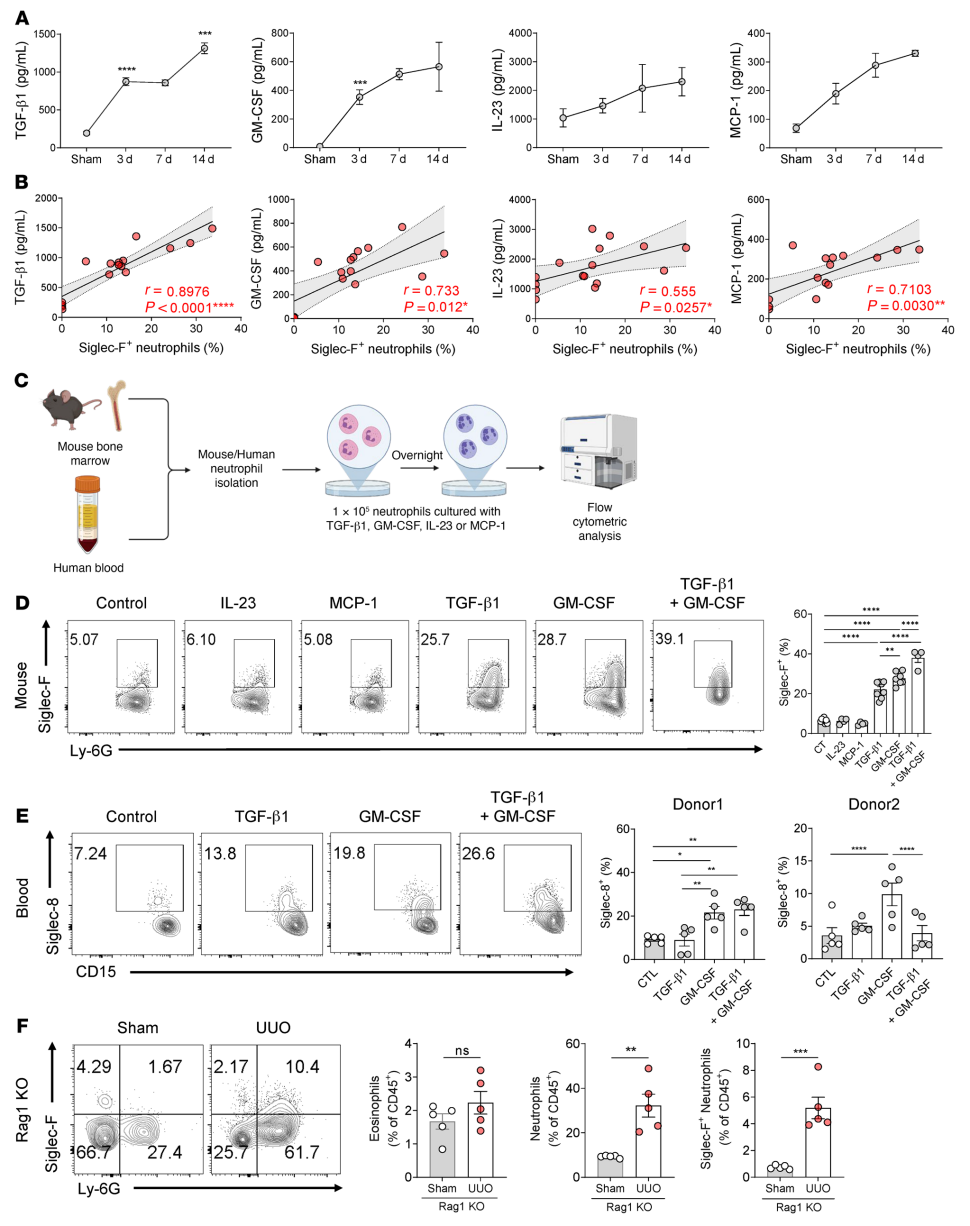
TGF-β1 and GM-CSF convert conventional neutrophils into Siglec-F–expressing neutrophils. (**A**) Multiplex cytokine bead array analysis of the levels of inflammatory cytokines in the kidney after UUO induction. (**B**) Pearson correlation analysis between the frequency of Siglec-F^+^ neutrophils and the upregulated cytokines shown in **A**. (**C**–**E**) Depiction of the experiment (**C**) where neutrophils were harvested from mouse bone marrow (**D**) or human blood (**E**); incubated overnight with 10 ng/mL of TGF-β1, GM-CSF, IL-23, and/or MCP-1; and then subjected to flow cytometric analysis of their Siglec-F (**D**) or Siglec-8 (**E**) expression. (**F**) Flow cytometric analysis of Siglec-F^+^ neutrophils in the kidney of *Rag1*^–/–^ mice 14 days after UUO induction. All results are shown as mean ± SEM, and statistical analysis was performed using 1-way ANOVA (**B**, **D**, and **E**) or Student’s *t* test (**F**). *P < 0.05; **P < 0.01; ***P < 0.001; ****P < 0.0001; *n* = 4–5 mice in each group.

To identify the factors that induced conventional neutrophils to convert into Siglec-F^+^ neutrophils after UUO, we examined the effect of UUO on the levels of tissue cytokines that are known to influence neutrophil plasticity (25, 26). Thus, the kidney lysates of sham- and UUO-treated mice were applied for multiplex cytokine bead arrays. This analysis showed that TGF-β1, GM-CSF, IL-23, and monocyte chemoattractant protein-1 (MCP-1) were rapidly upregulated soon after UUO (Figure 4A). This was not observed for IL-1α, IFN-γ, TNF-α, IL-12p70, IL-6, IL-17A, IFN-β, IL-27, IL-10, or IL-1β (Supplemental Figure 3). Correlation analyses also showed that TGF-β1, GM-CSF, IL-23, and MCP-1 levels correlated significantly and positively with the frequencies of Siglec-F^+^ neutrophils (Figure 4B).

To determine whether TGF-β1, GM-CSF, IL-23, and MCP-1 can directly induce conventional neutrophils to convert into Siglec-F^+^ neutrophils, we isolated neutrophils from murine bone marrow and then treated them with each cytokine in vitro (Figure 4C). Especially GM-CSF but also TGF-β1 (but not IL-23 or MCP-1) induced Siglec-F expression in neutrophils (Figure 4D). Moreover, when TGF-β1 and GM-CSF were combined, they had a small additive effect on Siglec-F^+^ neutrophil numbers in vitro (Figure 4D). To test whether these cytokines had similar effects on human neutrophils, neutrophils were isolated from the peripheral blood of 2 healthy blood donors and treated with TGF-β1 and/or GM-CSF (Figure 4C). Analysis of their expression of Siglec-8, which is a human counterpart of mouse Siglec-F (27), showed that GM-CSF (but not TGF-β1) induced Siglec-8^+^ neutrophils, although there were differences between the donors (Figure 4E).

T cells are well-known producers of TGF-β1 and GM-CSF; they are also known to play a pivotal role in renal fibrosis (28–30). To determine whether they are the source of the TGF-β1 and GM-CSF that causes conventional neutrophils to convert into Siglec-F^+^ neutrophils, we induced UUO in *Rag1*^–/–^ mice (T and B cell–deficient mice). However, Siglec-F^+^ neutrophils were still observed, although at a lower frequency than in WT mice (5% vs. 25% of CD45^+^ cells) (Figure 4F and Figure 2A). Thus, while T cells contribute some of these cytokines, it is likely that nonimmune cells, such as tubular epithelial cells (which are known to produce TGF-β1 and GM-CSF in renal fibrosis; refs. 31, 32), may also help induce Siglec-F^+^ neutrophils by generating inflammatory cytokines.

### Siglec-F^+^ neutrophils contribute to renal fibrosis by producing profibrotic factors that activate fibroblasts and by secreting collagen 1.

**Figure 5:**
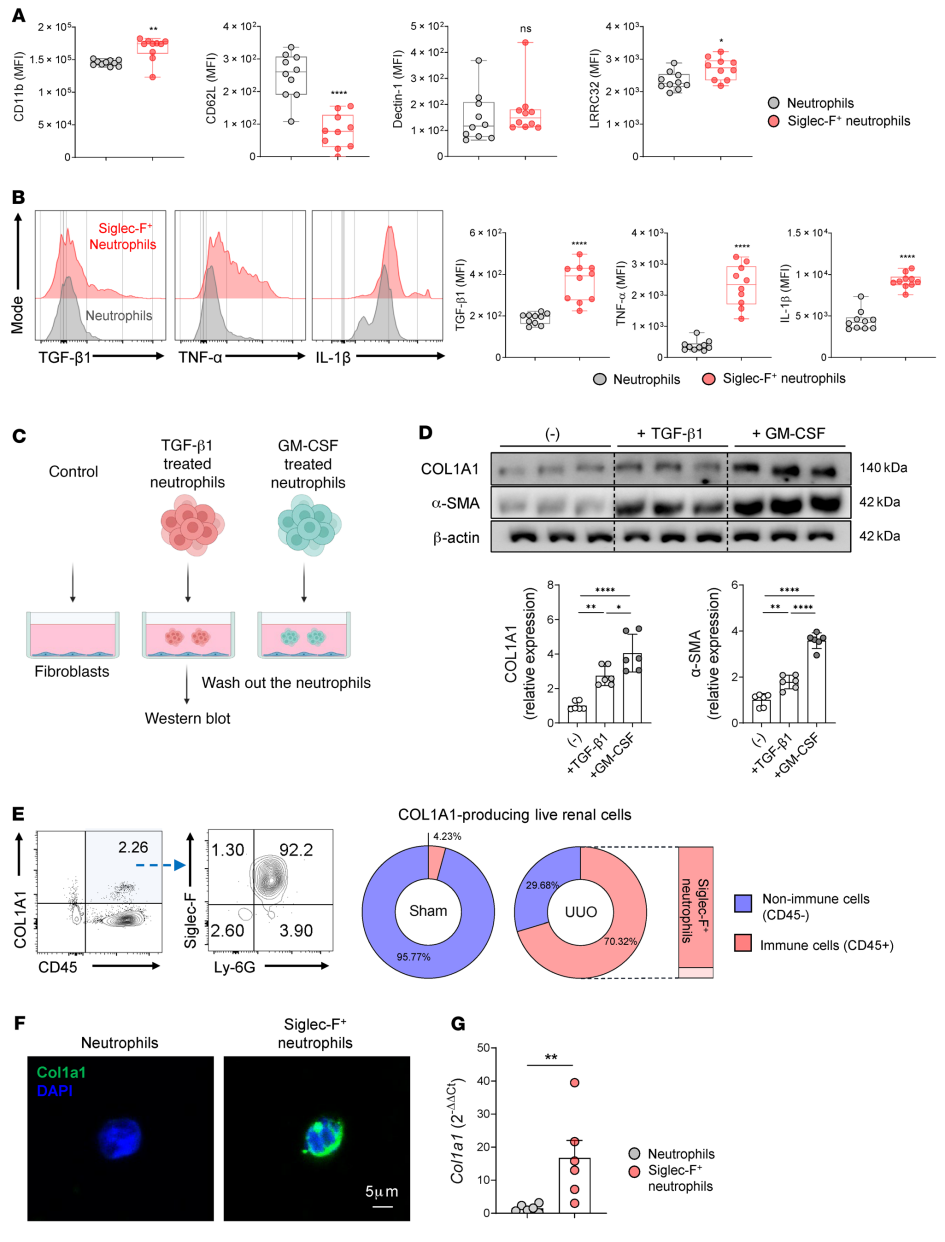
Siglec-F^+^ neutrophils produce more profibrotic cytokines than conventional neutrophils and are also collagen-producing cells. (**A** and **B**) Flow cytometric comparison of conventional neutrophils and Siglec-F^+^ neutrophils in the UUO-injured kidney at day 7 (*n* = 10 in each group) in terms of their surface (**A**) and intracellular (**B**) levels of inflammatory and homeostatic markers. The box plots depict the minimum and maximum values (whiskers), the upper and lower quartiles, and the median. The length of the box represents the interquartile range. (**C** and **D**) Depiction of the experiment (*n* = 6 in each group) where bone marrow–derived neutrophils were induced to convert into Siglec-F^+^ neutrophils by priming with TGF-β1 or GM-CSF, after which they were cocultured with NIH 3T3 fibroblasts (**C**). After the neutrophils were washed out, the fibroblasts were subjected to Western blot analysis for COL1A1 and α-SMA, which are fibroblast activation and differentiation markers (**D**). (**E**) Flow cytometric analysis of the intracellular levels of COL1A1 in the CD45^+^ immune cells and CD45^–^ non-immune cells from sham- and UUO-treated kidneys at day 7. (**F** and **G**) Sorted conventional and Siglec-F^+^ neutrophils from UUO-treated kidneys at day 14 (*n* = 6 in each group) were subjected to immunofluorescence (**F**) and RT-qPCR (**G**) analysis of COL1A1 protein expression. Scale bar: 5 μm. All results are shown as mean ± SEM, and statistical analysis was performed using Student’s *t* test (**A**, **B**, and **G**) or 1-way ANOVA (**D**). *P < 0.05; **P < 0.01; ****P < 0.0001.

We observed above that the Siglec-F^+^ neutrophils were more likely to display hypersegmentation than conventional neutrophils in the kidney after UUO (Figure 2C). Since neutrophil hypersegmentation is associated with distinct proteome and altered functions (33), we compared the conventional and Siglec-F^+^ neutrophils in the kidney after UUO for their expression of inflammatory and homeostatic markers (33, 34). Siglec-F^+^ neutrophils expressed significantly higher surface levels of CD11b and leucine-rich repeat-containing protein 32 (LRRC32) and lower levels of CD62L. LRRC32 converts the latent form of TGF-β1 into the active form (35). However, the 2 neutrophil populations did not differ in the surface levels of dectin-1 (Figure 5A), which is an inflammatory mediator that is upregulated in polarized neutrophils (34). Intracellular cytokine staining of proinflammatory cytokines then showed that Siglec-F^+^ neutrophils from the UUO-injured kidney expressed more TGF-β1, TNF-α, and IL-1β than conventional neutrophils (Figure 5B). This was confirmed by quantitative PCR (qPCR) analysis of the sorted neutrophils from UUO-injured kidneys (Supplemental Figure 4A).

Our in vitro experiment above showed that TGF-β1 and especially GM-CSF treatment converted murine bone marrow–derived neutrophils into Siglec-F^+^ neutrophils (Figure 4D). When we examined the levels of proinflammatory cytokines in these cells by flow cytometry, we found again that especially GM-CSF but also TGF-β1 treatment induced these cells to express the proinflammatory genes TGF-β1, TNF-α, and IL-1β (Supplemental Figure 4B). Since TGF-β1, TNF-α, and IL-1β are known to activate the fibrotic activities of fibroblasts (36, 37), these results together led us to hypothesize that the proinflammatory cytokines produced by Siglec-F^+^ neutrophils could induce fibroblast activation and differentiation. To test this, we cocultured in vitro unprimed (conventional) neutrophils or TGF-β1– or GM-CSF–primed neutrophils with NIH 3T3, a mouse embryonic fibroblast cell line. After 24 hours, we washed out the neutrophils and subjected the fibroblasts to Western blot analysis of 2 fibroblast activation and differentiation markers, namely, COL1A1 and α-SMA (Figure 5C). Both were elevated in the fibroblasts that had been cultured with the primed neutrophils. Moreover, in line with the greater effect of GM-CSF on the neutrophil expression of Siglec-F (Figure 4D), the GM-CSF–primed neutrophils activated the fibroblasts more potently than the TGF-β1–primed neutrophils (Figure 5D).

Recent studies have shown that fibroblasts are not the only cells that can produce collagen 1 in renal fibrosis: immune cells, specifically macrophages, also have this capacity (38, 39). Indeed, when we enumerated the COL1A1-expressing CD45^+^ immune cells and CD45^–^ nonimmune cells from sham- and UUO-treated kidneys by flow cytometry, we found that UUO was associated with a huge increase in COL1A1-producing CD45^+^ immune cells (from 4% in the sham-treated mice to 70% in the UUO-treated mice) (Figure 5E). An analysis of the immune cell types then showed that the vast majority of the COL1A1-expressing CD45^+^ leukocytes after UUO were Siglec-F^+^ neutrophils (Figure 5E). We then confirmed with immunofluorescence staining and qPCR that Siglec-F^+^ neutrophils isolated from UUO kidneys expressed COL1A1; by contrast, the conventional neutrophils did not express COL1A1 (Figure 5, F and G). Similarly, the bone marrow–derived neutrophils that had been primed in vitro with GM-CSF exhibited elevated COL1A1 expression compared with the unprimed neutrophils (Supplemental Figure 4, C and D). Thus, Siglec-F^+^ neutrophils may contribute to renal fibrosis by 2 mechanisms, namely, by activating fibroblasts via their profibrotic cytokines (TGF-β1, TNF-α, and IL-1β) and by directly secreting COL1A1.

### Siglec-F^+^ neutrophils contribute to advanced renal fibrosis.

**Figure 6:**
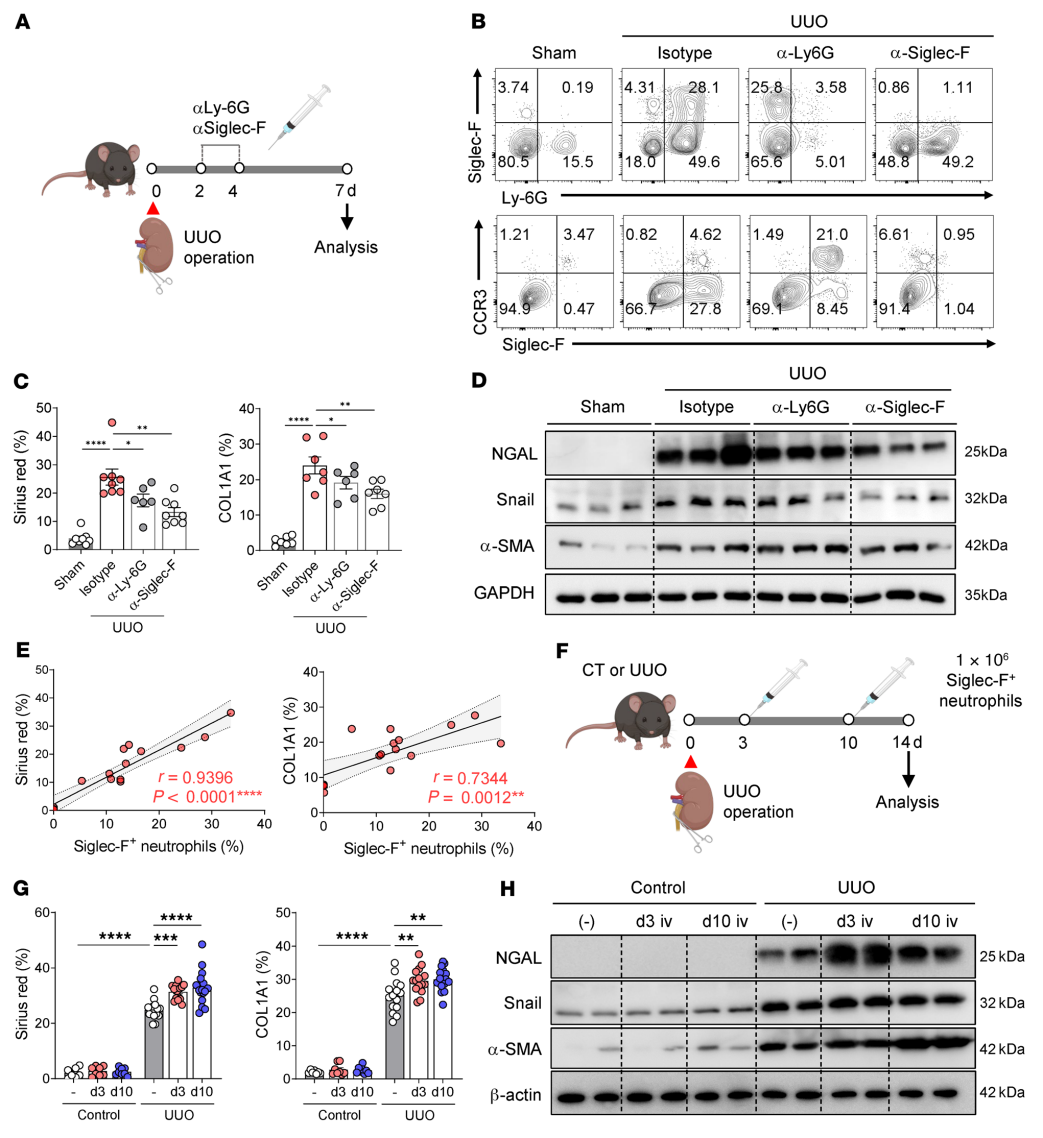
Siglec-F^+^ neutrophils promote immunopathology during UUO-induced renal injury. (**A**) UUO mice were treated on days 2 and 4 with isotype control, anti–Ly-6G, or anti–Siglec-F mAbs. (**B**) The anti–Ly-6G and anti–Siglec-F mAbs effectively depleted the Siglec-F^+^ neutrophils in the kidney; the anti–Ly-6G antibodies also depleted the conventional neutrophils in the kidney. (**C**, **D**, **G**, and **H**) The effect of Siglec-F^+^ neutrophil depletion (**C** and **D**) or transfer (**G** and **H**) on the degree of renal injury was determined on day 7 (depletion) or day 14 (transfer) by Sirus red histology and COL1A1 IHC and Western blotting of kidney damage– and fibrosis-related markers. (**E**) Pearson correlation analysis between Siglec-F^+^ neutrophil frequencies and the percentage of Sirius red staining and COL1A1 staining in the UUO-damaged kidneys. (**F**) Siglec-F^+^ neutrophils were adoptively transferred into the control or UUO mice on days 3 or 10 from the injury. All results are shown as mean ± SEM, and statistical analysis was performed using 1-way ANOVA. *P < 0.05; **P < 0.01; ***P < 0.001; ****P < 0.0001; *n* = 6–7 mice in each group for **A**–**D**, *n* = 7–16 mice in each group for **F**–**H**.

To evaluate whether Siglec-F^+^ neutrophils play an essential role in renal fibrosis, we depleted Siglec-F^+^ neutrophils with doses of anti–Siglec-F or anti–Ly-6G antibodies on days 2 and 4 after UUO (Figure 6A). Flow cytometry on day 7 showed that the anti–Ly-6G antibodies depleted both Siglec-F^+^ neutrophils and conventional neutrophils in the kidney, whereas the anti–Siglec-F antibodies specifically removed the Siglec-F^+^ neutrophils (Figure 6B). Renal fibrosis was measured on day 7 with Sirius red histology and COL1A1 IHC. Although both antibodies reduced fibrosis, the anti–Siglec-F antibody was more effective (Figure 6C and Supplemental Figure 5A). It also reduced the kidney expression of renal damage– and fibrosis-related markers better than the anti–Ly-6G antibody (Figure 6D and Supplemental Figure 5B). This difference may partly reflect the large increase in Siglec-F^+^ CCR3^+^ eosinophils in the UUO mice treated with anti–Ly-6G (25.8% vs. 4.3% in the isotype-treated mice and 0.9% in the mice treated with anti–Siglec-F) (Figure 6B), which could potentially promote fibrosis. Consistent with these results, the Siglec-F^+^ neutrophil frequencies correlated highly significantly and positively with the degree of renal fibrosis, as quantified by Sirius red and COL1A1 staining (Figure 6E).

To further determine whether Siglec-F^+^ neutrophils directly induce renal fibrosis, we adoptively transferred in vitro–generated Siglec-F^+^ neutrophils into control and UUO mice (Figure 6F). In UUO mice, renal fibrosis was significantly enhanced by the delivery of Siglec-F^+^ neutrophils (Figure 6, G and H, and Supplemental Figure 5, C and D); however, the adoptive transfer of Siglec-F^+^ neutrophils into control mice did not increase renal fibrosis. Indeed, transferred Siglec-F^+^ neutrophils were barely found in the kidneys of control mice (Supplemental Figure 5F), suggesting that i.v. transferred Siglec-F^+^ neutrophils did not migrate to the uninjured kidney. Taken together, these results support the notion that Siglec-F^+^ neutrophils play a crucial role in renal fibrosis.

### Siglec-F^+^ neutrophils are increased in the kidneys of other murine CKD models and human kidneys with renal cell carcinoma.

**Figure 7:**
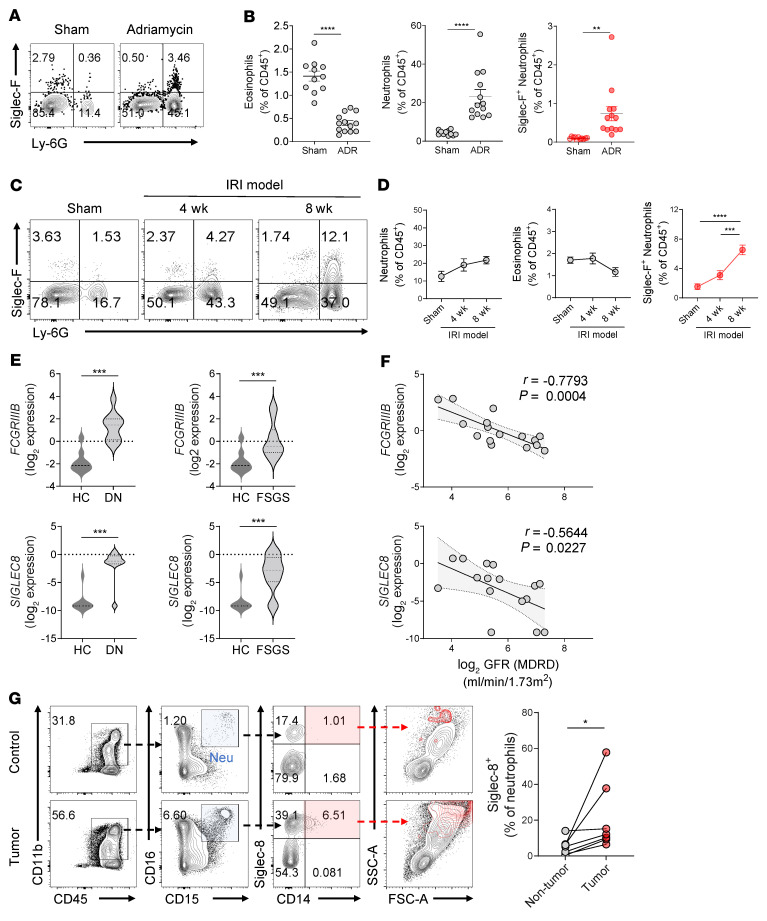
Siglec-F^+^ neutrophils are generated in other mouse fibrosis models and in human kidneys with fibrotic changes. (**A**–**D**) Adriamycin-induced nephropathy (**A** and **B**) (*n* = 11–13 mice in each group) and renal ischemia/reperfusion injury (IRI) (**C** and **D**) (*n* = 8 mice in each group) were induced and evaluated on day 7 and at 4 or 8 weeks, respectively. The changes in eosinophil, neutrophil, and Siglec-F^+^ neutrophil frequencies were determined by flow cytometry (**B** and **D**). (**E** and **F**) A public gene expression data set of patients with CKD was used to determine whether diabetic nephropathy (DN) (*n* = 9) and focal segmental glomerulosclerosis (FSGS) (*n* = 19) were associated with increased renal frequencies of neutrophils (as indicated by the *FCGRIIIB* gene) and Siglec-8^+^ neutrophils (as indicated by the *SIGLEC8* gene) compared with the healthy control (HC) group (*n* = 10) (**E**). Pearson correlation analysis between *FCGRIIIB* and *SIGLEC8* expression and renal function (log_2_ GFR) in patients with DN (*n* = 16) was also assessed (**F**). (**G**) Nephrectomy specimens from 7 patients with clear cell renal cell carcinoma were subjected to flow cytometry to determine the frequencies of Siglec-8^+^ neutrophils (CD11b^+^CD15^+^CD16^+^ and SSC^hi^) in the healthy and tumor counterparts of each specimen. All results are shown as mean ± SEM, and statistical analysis was performed using Student’s *t* test (**A** and **E**), 1-way ANOVA (**B**), or Wilcoxon rank-sum test (**G**). *P < 0.05; **P < 0.01; ***P < 0.001; ****P < 0.0001.

Finally, we investigated whether Siglec-F^+^ neutrophils also contribute to fibrosis progression in CKD models caused by other etiologies. One was Adriamycin-induced nephropathy, which serves as the focal segmental glomerulosclerosis (FSGS) model and has the advantage that it induces glomerular and tubular interstitial fibrosis (ref. 40 and Supplemental Figure 6A). Like the UUO model, Adriamycin-induced nephropathy is associated with increased kidney frequencies of Siglec-F^+^ neutrophils (Figure 7, A and B). Since UUO and Adriamycin nephropathy are models of acute renal fibrosis, we also examined the renal ischemia/reperfusion injury (IRI) model, which has the advantage of transitions from acute kidney injury to CKD (41). As expected, fibrosis arose slowly in the IRI model (Supplemental Figure 6B). However, a significant increase in Siglec-F^+^ neutrophil frequency was observed 4 and 8 weeks after the kidney injury (Figure 7, C and D). Thus, Siglec-F^+^ neutrophils may participate in various renal diseases characterized by fibrosis.

To expand our findings from mouse CKD models to human renal fibrosis, we analyzed a public gene expression database of patients with CKD (Nephroseq Research Edition, available from http://www.nephroseq.org). Since humans lack the Siglec-F gene, we instead analyzed Siglec-8, a paralog of Siglec-F. Compared to healthy renal tissues, the renal tissues of patients with diabetic nephropathy and FSGS exhibited significantly increased expression of both *FCGRIIIB* (a surrogate marker of neutrophils) and *SIGLEC8* (Figure 7E). In addition, the expression of both genes in patients with diabetic nephropathy correlated significantly and negatively with their renal function, namely, the log_2_ glomerular filtration rate (GFR) (Figure 7F). These results suggest that Siglec-8^+^ neutrophils increase in patients with CKD and may be associated with disease exacerbation.

Since intratumoral fibrosis is a common feature of clear cell renal cell carcinoma (42), we also analyzed the Siglec-8^+^ neutrophils in the renal tissues of 7 patients with clear cell renal cell carcinoma. Indeed, we confirmed that the tumor tissues exhibited elevated fibrosis and collagen deposition compared with the normal kidney tissues in the surgical specimens (Supplemental Figure 6C). Analysis of the healthy and tumor kidney tissues of the same donors then showed that Siglec-8^+^ neutrophils were only present in the tumor fractions (Figure 7G). These data together support the notion that this neutrophil subset contributes to renal fibrosis in not only murine kidneys but also in human kidneys.

## Discussion

Given that neutrophils are the most abundant inflammatory cells in the blood, extensive research has been conducted on not only their protective roles against infections but also their pathogenic activities in various inflammatory and fibrotic diseases (43, 44). However, their roles in renal fibrosis have not received much attention. Here, we report that a recently described Siglec-F–expressing neutrophil subset was elicited and strongly expanded in the fibrotic kidney after ureteral obstruction. Our subsequent comprehensive characterization of these cells then showed that although they are derived from conventional neutrophils, they differ from their parent cells in that they are larger and more granular, are more likely to be hypersegmented, and express higher levels of the CD11b and LRRC32 activation markers and the TGF-β1, TNF-α, and IL-1β effector cytokines. Moreover, unlike conventional neutrophils, Siglec-F–expressing neutrophils effectively induced fibroblasts to produce collagen 1; this effect was mediated by the molecules secreted by the Siglec-F–expressing neutrophils. Importantly, we also showed that these cells produce collagen 1. This suggests Siglec-F–expressing neutrophils contribute to fibrosis by a) directly producing collagen 1 and b) inducing other cells to produce collagen 1. In addition, we observed that Siglec-F^+^ neutrophils were also elicited in other models of CKD (i.e., Adriamycin-induced nephropathy and IRI). Moreover, *SIGLEC8*, the human counterpart of Siglec-F, was upregulated in renal tissues from patients with diabetic nephropathy or FSGS compared with healthy controls as well as in fibrotic renal tumor tissues from patients compared with their healthy renal tissues. Finally, depletion and adoptive transfer of Siglec-F^+^ neutrophils clearly demonstrated their involvement in renal fibrosis and suggested them as potential therapeutic targets. Since Siglec-F and Ly-6G are, respectively, eosinophil and neutrophil markers, our initial experiments after finding the Siglec-F^+^Ly-6G^+^ cells in fibrotic kidneys assessed whether these cells belonged to the neutrophil or eosinophil lineage. They were found to be neutrophils since a) they bore the characteristic blue Diff-Quik–stained multilobed nuclei that characterize neutrophils; b) they coexpressed the neutrophil marker Siglec-E but not the eosinophil marker CCR3; c) they were also induced by UUO in ΔdblGATA mice, which cannot generate eosinophils; and d) they could be produced in vitro from naive neutrophils by culture with GM-CSF or TGF-β1.

Siglec-F^+^ neutrophils were first identified in a 2017 study on tumors (12). Their tumor-promoting role has since been observed in other studies as well (13, 16). Siglec-F^+^ neutrophils have also been reported to be elicited in several other inflammatory conditions, namely, nasal infection (15), allergic rhinitis (17), asthma (19), and myocardial infarction (18). Unlike our study, the tumor studies found that the Siglec-F^+^ neutrophils did not differ morphologically from conventional neutrophils (12, 13). It also seems that the role of Siglec-F^+^ neutrophils depends on the context. Specifically, while Siglec-F^+^ neutrophils in tumors promote tumor growth regardless of the type of tumor (16), these cells conversely also support the repair of the inflamed olfactory neuroepithelium (17) and the clearance of *Bordetella pertussis* from the nasal epithelium (15). These observations suggest that while Siglec-F^+^ neutrophils can be generated by various conditions, their functions may be shaped by the specific stimuli and microenvironments in which they are formed and function. Further studies on the effect of context on Siglec-F^+^ neutrophil activities are warranted.

We observed that Siglec-F^+^ neutrophils were not present in the peripheral blood, spleen, or bone marrow of the UUO mice. The rapid growth of the Siglec-F^+^ neutrophil population in the injured kidney was accompanied by an equally dramatic increase of conventional neutrophils in the peripheral blood, spleen, and damaged kidney. This suggests that Siglec-F^+^ neutrophils in the fibrotic kidney are generated from conventional neutrophils in either the parenchyma of the damaged kidney or its renal vasculature. Our BV650-CD45 mAb injection experiments showed that the latter was true. Thus, in the sham-treated mice, the few Siglec-F^+^ neutrophils in the healthy kidney were nearly all in the renal vessels. After UUO, however, half of the rapidly expanding Siglec-F^+^ neutrophil population in the injured kidney was located in the parenchyma, with the other half being in the blood vessels. Since this pattern was still observed 14 days after UUO and the Siglec-F^+^ neutrophils in the injured kidney were also poorly proliferative and apoptotic, it seems that the parenchymal Siglec-F^+^ neutrophil population is constantly being replenished by conversion of conventional neutrophils in the renal vessels, after which the newly formed Siglec-F^+^ neutrophils migrate into the renal parenchyma.

Many lines of evidence suggest that inflammatory cytokines play a critical role in the initiation and progression of CKD (45). We observed that the UUO-injured kidneys of our mice expressed higher levels of TGF-β1, GM-CSF, IL-23, and MCP-1 compared with the sham-treated kidneys. We therefore hypothesized that this altered cytokine milieu in the kidney could drive the generation of Siglec-F^+^ neutrophils. Indeed, the kidney levels of these cytokines after UUO correlated positively with Siglec-F^+^ neutrophil frequencies in the kidney. Moreover, treatment with TGF-β1 and GM-CSF induced Siglec-F expression in cultured conventional murine neutrophils and Siglec-8 expression in naive human neutrophils. Thus, TGF-β1 and particularly GM-CSF cause conventional neutrophils to convert into Siglec-F^+^ neutrophils.

Of note, we found that the Siglec-F^+^ neutrophils generated after UUO or by TGF-β1 or GM-CSF priming expressed collagen 1. It is conventionally thought that myofibroblasts produce most of the extracellular matrix protein in renal fibrosis (46). This was disputed recently by the study of Buchtler et al., who examined the degree of renal fibrosis in mice in which *Col1a1*-expressing CD45^+^ cells have been conditionally knocked out: when these mice were subjected to UUO or Adriamycin-induced nephropathy, their renal fibrosis was significantly reduced compared with WT mice (38). Although they did not specify which CD45^+^ leukocytes were producing the collagen 1, they concluded that leukocytes contribute 38% to 50% of collagen 1 deposition in renal fibrosis. This is supported by a recent study that reanalyzed the single-cell RNA-Seq data from UUO-injured kidneys (47): it showed that immune cells, including neutrophils, from injured kidneys express *Col1a1* as well as other collagen proteins such as *Col5a2*, *Col12a1*, and *Col15a1* (48). Our observation that Siglec-F^+^ neutrophils are the major collagen 1–producing leukocytes in the fibrotic kidney is thus consistent with and expands these findings. It is also supported by our Siglec-F^+^ neutrophil mAb-depletion experiments: both the anti–Ly-6G and anti–Siglec-F mAbs significantly reduced UUO-induced fibrosis. Notably, we observed that although both mAbs efficiently depleted Siglec-F^+^ neutrophils, the anti–Ly-6G reduced fibrosis less effectively than the anti–Siglec-F mAb. This may reflect the unexpected increase of eosinophils after anti–Ly-6G treatment, which could have profibrotic potential. Further mechanistic studies are required.

Although the contribution of neutrophils to renal failure has been relatively poorly studied to date, a few studies clearly show they do exist and participate in glomerulonephritis with diverse etiologies (49–52). The present study is consistent with and expands these findings by showing that the profibrotic Siglec-F^+^ neutrophil was elicited in fibrotic kidneys in both murine models and humans. This suggests that neutrophil profiling in CKD may be of clinical value. Moreover, our data suggest that this neutrophil subtype could be targeted by immunotherapies, thereby preventing CKD or halting its inexorable progression. To determine the potential clinical applicability of our findings, it will be necessary to first verify the presence of Siglec-8–expressing neutrophils and their characteristics at specific CKD stages in large and diverse patient cohorts. Nonetheless, from the pathophysiological point of view, we anticipate that the functional analysis and molecular profiling of the Siglec-F^+^ neutrophils that have been described here will help the field gain new insights into the microinflammatory environment that is associated with CKD.

One of the limitations of our study is we could not directly quantify how much the 2 profibrotic mechanisms of Siglec-F^+^ neutrophils contribute to renal fibrosis: Does their production of collagen 1 play a bigger role or is it rather their production of profibrotic cytokines (which induces other cells to produce collagen 1)? The use of the *Col1a1* conditional knockout system in neutrophils may provide an answer to these questions. In addition, we have not yet been able to examine renal tissues from patients with CKD to verify that these tissues also bear Siglec-8^+^ neutrophils and that they contribute to disease progression. Although these studies are in progress, they are hampered by difficulties in obtaining sufficient kidney tissues from patients with CKD for flow cytometric analysis. Nevertheless, our study showed that Siglec-F^+^ or Siglec-8^+^ neutrophils are indeed present in the fibrotic kidney. Our comprehensive analysis with various models of CKD, in vitro experiments, and human gene and tissue experiments also suggests that these cells contribute significantly to the fibrotic process and could be targeted by therapeutic strategies that promote tissue recovery and minimize the progression of CKD and perhaps also other forms of fibrosis.

## Methods

### Animal models.

Six- to 10-week-old C57BL/6 male mice, ΔdblGATA, or *Rag1*^–/–^ mice were subjected to UUO, IRI, or sham operation. The UUO model was induced by ligating the left ureter with 5-0 silk at 2 points. The kidneys were excised 3, 7, and 14 days after surgery. The IRI model was induced by clamping the left renal pedicle with a microvascular clamp (Roboz Surgical Instrument) for 30 minutes. The mice were euthanized 4 and 8 weeks after surgery. Sham operations were identical to UUO, Adriamycin treatment, or IRI except that the ureter or renal artery were not manipulated or the mice were injected with PBS instead of Adriamycin. Eight-week-old BALB/c male mice were subjected to UUO operation or Adriamycin-induced nephropathy. UUO was conducted as described above. The Adriamycin-induced model was established with an i.v. injection of Adriamycin (11.5 mg/kg mouse weight; Tokyo Chemical Industry). The mice were euthanized 7 days after the induction. All WT mice were purchased from Koatech. The *Rag1*^–/–^ [B6.129S7-*Rag1^tm1Mom^*/J (stock 002216)] and ΔdblGATA [C129S1(B6)-*Gata1^tm6Sho^*/J (stock 005653)] mice were purchased from the Jackson Laboratory. All mice used in the study were maintained in the specific pathogen–free animal facility at the Seoul National University Hospital Biomedical Research Institute, Korea, and were used under approved study protocol 20-0255.

### Human samples.

Seven patients who underwent nephrectomy for renal cell carcinoma at the Seoul National University Hospital provided renal tissues. Tissues were evaluated by the pathologist and divided into the normal and tumor parts. Peripheral blood was also collected from 2 healthy donors. All biospecimens were provided by the Seoul National University Human Biobank, which is a member of the National Biobank of Korea and is supported by the Ministry of Health and Welfare (Korea).

### Histological, immunohistological, cytological, and IHC analyses.

FFPE tissue blocks were generated from experimental mouse kidneys or human kidney tissues. Tissue sections (4 μm thick) were deparaffinized and rehydrated before the staining. The sections were subjected to histological analysis by staining with periodic acid–Schiff (PAS), Masson’s trichrome (MT), or Sirius red. Collagen 1 levels in the sections were also determined by performing antigen retrieval and incubating the sections first with the primary antibody for COL1A1 (1:100; Santa Cruz Biotechnology, catalog sc-293182) and then HRP-conjugated secondary antibodies (Dako, code K4001) according to the manufacturer’s instructions. To quantify fibrosis in the kidneys, 10 fields were randomly selected under ×100 original magnification and the amount of Sirius red staining and COL1A1 levels were determined. To characterize neutrophil morphology, neutrophils sorted as described further below were centrifuged with a cytocentrifuge, fixed with 100% methanol, and stained with Diff-Quik (Sysmex) according to the manufacturer’s instructions. All images were acquired with a Leica DM750 microscope and analyzed with Leica LasX software.

### Tissue protein analysis with Western blot and bead array.

Protein from experimental mouse kidney tissues was prepared with RIPA buffer containing protease inhibitor cocktail and EDTA. The amount of total protein was quantified using the bicinchoninic acid assay (Pierce BCA protein assay kit, Thermo Fisher Scientific) and adjusted to a concentration of 1 to 2 mg/mL. For Western blot analysis, 80 μg of extracted proteins were run on 8%–12% gradient gels and transferred onto Immobilon PVDF membranes (Merck Millipore). Antibodies used for immunoblotting were as follows: NGAL (1:100, Santa Cruz Biotechnology, sc-515876), P16 (1:500, MilliporeSigma, LS-B5261), COL1A1 (1:1000, Novus, NBP1-30054), α-SMA (1:1000, Abcam, ab32575), fibronectin (1:500, Abcam, ab2413), Snail (1:400; Abcam, ab180714), β-actin (1:10,000, MilliporeSigma, a1978), and GAPDH (1:5000, MilliporeSigma, 14c10). Images were acquired by a gel documentation system (Gel Doc 1000, Bio-Rad). Protein bands from Western blot were quantified with ImageJ (NIH). To screen for the proinflammatory cytokines that converted conventional neutrophils into Siglec-F^+^ neutrophils, a bead-based immunoassay was performed (LEGENDplex mouse inflammation panel and active/total TGF-β1 assay, BioLegend) according to the manufacturer’s instructions. The array was analyzed by BD Biosciences LSRFortessa X-20 flow cytometer and web-based software provided by the manufacturer (https://legendplex.qognit.com).

### Isolation of single human/mouse kidney leukocytes.

Whole kidneys from experimental mice or portions of nephrectomized kidneys from patients were freshly prepared in cold PBS. Tissues were mechanically dissociated into small pieces using a scalpel blade. To further dissociate the cells, the tissues were subjected to enzymatic digestion with 1 mg/mL collagenase IV (Worthington Biochemical) and 50 μg/mL DNase I (MilliporeSigma) for 45 minutes at 37°C. After digestion, the cells were filtered with 40 μm strainers and washed with cold PBS. The cell pellet was then resuspended in 40% Percoll solution (GE Healthcare) and layered on 80% Percoll solution. The leukocyte layers between the 2 Percoll solutions were obtained after centrifugation at 800*g* at 4°C. The leukocytes were washed for further analysis.

### Flow cytometric analysis, cell sorting, and subsequent analyses.

Single mouse or human cell preparations were resuspended in PBS with Zombie-Aqua viability dye (BioLegend) and mouse or human Fc block (BD Biosciences) and incubated for 10 minutes. For surface marker staining, cells were resuspended in FACS buffer (PBS with 2% FBS) and incubated for 30 minutes with the following FITC-, PerCP-Cy5.5–, PE-Cy7–, PE-, APC-, BV421-, BV650-, or BV785-conjugated mouse or human mAbs. The mouse antibodies were specific for I-Ab (AF6-120.1, BioLegend), Ly-6G (1A8, BioLegend), Siglec-E (M1304A01, BioLegend), CCR3 (J073E5, BioLegend), CD11c (HL3, BD Biosciences), CD11b (M1/70, BioLegend), F4/80 (BM8, BioLegend), Siglec-F (S17007L, BioLegend), CD19 (1D3, BD Biosciences), CD3e (145-2C11, BioLegend), NK1.1 (PK136, BioLegend), CD8a (53-6.7, BioLegend), CD90.2 (30-H12, BioLegend), CD25 (PC61, BioLegend), CD4 (RM4-5, BioLegend), and CD45 (30-F11, BioLegend). The human antibodies were specific for CD14 (HCD14, BioLegend), CD15 (W6D3, BioLegend), Siglec-8 (7C9, BioLegend), CD16 (3G8, BioLegend), and CD45 (HI30, BioLegend). For the annexin V assays, the cells were incubated for 30 minutes in annexin V buffer with annexin V (BioLegend) and analyzed within 30 minutes according to the manufacturer’s instructions. For the proliferation and intracellular staining assays, cells were fixed and permeabilized with eBioscience Foxp3/transcription factor staining kit (Invitrogen) according to the manufacturer’s instructions. The cells were then incubated for an hour in Foxp3/transcription factor staining buffer with Ki-67 (BioLegend) or COL1A1 (polyclonal, Rockland Immunochemicals). For intracellular cytokine staining, cells were restimulated with 100 ng/mL LPS (Merck) for 3 hours. After fixation and permeabilization with BD Biosciences Cytofix/Cytoperm kit according to the manufacturer’s instructions, the cells were incubated with antibodies specific for TGF-β1 (TW7-16B4, BioLegend), TNF-α (MP6-XT22, Invitrogen), and IL-1β (NJTEN3, BD Biosciences). Samples were analyzed using the BD Biosciences LSRFortessa X-20 flow cytometer, and the data were processed using FlowJo (v10.6.1., BD Biosciences). To obtain neutrophils that did and did not express Siglec-F, cells were stained with the flow cytometric analysis antibodies described above. The cells were sorted as live (Zombie-Aqua^–^), CD45^+^CD11b^+^Ly-6G^+^, and Siglec-F positive or negative with BD Biosciences FACSAria III cell sorter. The cells were subjected to morphological assessment using the Diff-Quik staining kit (Sysmex), immunofluorescence imaging of COL1A1, and real-time qPCR as follows.

### Real-time qPCR.

RNA from tissues or sorted cells was extracted with TRIzol reagent (Invitrogen) according to the manufacturer’s protocol. cDNA was synthesized from 1 μg of RNA using the SensiFAST cDNA synthesis kit (Bioline). The cDNA was used as templates for qPCR using the SensiFAST SYBR Lo-ROX kit or Probe Lo-ROX kit (Bioline). qPCR was performed using the CFX96 Real-Time PCR Detection System (Bio-Rad) according to the manufacturer’s instructions. The target gene expression was normalized by *Gapdh* level in each sample. The sequences of the primers used in the study were the following: *Il1b* forward: 5′ TCGCTCAGGGTCACAAGAAA 3′, reverse: 5′ CATCAGAGGCAAGGAGGAAAAC 3′; *Tnfa* forward: 5′ GGTGCCTATGTCTCAGCCTCTT 3′, reverse: 5′ GCCATAGAACTGATGAGAGGGAG 3′; *Tgfb1* forward: 5′ TTGCCGAGGGTTCCCGCTCT 3′, reverse: 5′ CCTCCCGGGCGTCAGCACTA 3′; *Col1a1* forward: 5′ CCTGGTAAAGATGGTGCC 3′, reverse: 5′ CACCAGGTTCACCTTTCGCACC 3′.

### Expansion of eosinophil populations with IL-33 injections.

To test whether the Siglec-F^+^ neutrophils could be eosinophils, the UUO mice were i.p. injected with 250 ng recombinant IL-33 (BioLegend) on postsurgical days 0, 1, 2, and 3.

### Labeling of leukocytes in the renal vasculature.

To determine whether Siglec-F^+^ neutrophils arose in the kidney vasculature or parenchyma, sham- and UUO-treated mice were i.v. injected with a BV650-labeled CD45 mAb (BioLegend) 5 minutes before euthanasia.

### Siglec-F^+^ neutrophil depletion with anti–Siglec-F and anti–Ly-6G antibodies.

To test the role of Siglec-F^+^ neutrophils in CKD, UUO mice were depleted of these cells by injecting Siglec-F or Ly-6G depletion antibodies. On days 2 and 4 after surgery, mice were i.p. injected with the isotype antibody (40 μg/200 μL PBS; KLH, R&D Systems), the Siglec-F depletion antibody (40 μg/200 μL PBS; Q920G3, R&D Systems), or the Ly-6G depletion antibody (400 μg/200 μL; 1A8, BioXCell). Flow cytometric analysis was performed to evaluate the degree of depletion by the treatments.

### In vitro neutrophil conversion study and coculture of neutrophils and fibroblasts.

Neutrophils were isolated from naive mouse bone marrow or human blood by using the magnetic-activated cell sorting kit for mouse or human neutrophil isolation (Miltenyi Biotec). The purity of the isolated neutrophils exceeded 90%. The freshly prepared neutrophils (1 × 10^5^ cells/well) were incubated overnight in a 96-well plate with RPMI 1640 medium (biowest) containing 10% FBS and 10 ng/mL of the recombinant proteins IL-23 (BioLegend), MCP-1 (BioLegend), TGF-β1 (BioLegend), and/or GM-CSF (BioLegend). After washing with PBS, the cells were analyzed with flow cytometry for Siglec-F expression or ELISA (Cusabio Technology) and immunofluorescence imaging for COL1A1 expression. For the coculture study, 3 × 10^5^ NIH 3T3 fibroblasts/well (ATCC) were plated in a 6-well plate and cultured in serum-starved RPMI 1640 medium a day before coculture. Neutrophils that had been primed in vitro with TGF-β1 or GM-CSF were then added (3 × 10^6^/well). After coculture for 24 hours, the neutrophils were washed out with 3 PBS washes, and the fibroblast lysates were subjected to Western blot to assess the amounts of COL1A1 and α-SMA.

### Adoptive transfer of Siglec-F^+^ neutrophils.

Siglec-F^+^ neutrophils were prepared from the in vitro conversion with TGF-β1 and GM-CSF of bone marrow–derived neutrophils as described above. Control or UUO mice were i.v. injected with 1 × 10^6^ cells of Siglec-F^+^ neutrophils after 3 or 10 days of UUO. All mice were euthanized at day 14 of UUO and investigated for renal inflammation and fibrosis using histological analysis, Western blot, and RT-PCR.

### Statistics.

The data are presented as mean ± SEM. Depending on the normality of the data, groups were compared using paired/unpaired 2-tailed Student’s *t* test, Mann-Whitney *U* test, Wilcoxon rank-sum test, or 1-way ANOVA followed by Dunnett’s post hoc test. Correlation analyses with parametric or nonparametric data were conducted with Pearson’s or Spearman’s correlation test, respectively. The significance level (*P* value) for 2-tailed tests was 0.05. Statistical analysis was performed with GraphPad Prism 9.

### Study approval.

Animal studies were approved by the Seoul National University Hospital IACUC (protocol 20-0255). To assess the clinical implications of our findings regarding Siglec-F^+^ neutrophils in the animal studies, we also analyzed the immune cell populations in healthy and tumor tissues from patients. All study participants provided written informed consent. The study protocol was approved by the Seoul National University Hospital IRB (IRB 2106.081.1226).

## Author contributions

SHY and HYK conceptualized the study. SR, SHY, and HYK contributed to methodology. SR, JWS, SK, JL, Yoe-Sik Bae, Yong-Soo Bae, YCK, DKK, YSK, SHY, and HYK conducted the investigation. SR, SHY, and HYK performed visualization. SR, SHY, and HYK acquired funding. SR, SK, and JL contributed to project administration. SHY and HYK supervised the study. SR, SHY, and HYK wrote the original draft. SHY and HYK reviewed and edited the manuscript.

## Supplementary Material

Supplemental data

## Version 1 04/28/2022

In-Press Preview

## Version 2 06/15/2022

Electronic publication
