# Supplementary material for: Siglec-F–expressing neutrophils are essential for creating a profibrotic microenvironment in renal fibrosis
Source: J Clin Invest. 2022 Jun 15;132(12):e156876. doi: 10.1172/JCI156876 (PMC9197522; doi:10.1172/JCI156876)
Supplement: Supplemental data [file jci-132-156876-s008.pdf]

Supplementary Materials for

Siglec-F-expressing neutrophils are essential for creating a pro-fibrotic microenvironment in the obstructed kidney

Seungwon Ryu, Jae Woo Shin, Soie Kwon, Jiwon Lee, Yong Chul Kim, Yoe-Sik Bae, Yong-Soo Bae, Dong Ki Kim, Yon Su Kim, Seung Hee Yang*, Hye Young Kim*

*Corresponding authors. Email: ysh5794@gmail.com (SHY), hykim11@snu.ac.kr (HYK)

This PDF file includes:

Figure S1. Inflammation and fibrosis evaluation in UUO-induced kidney injury.
Figure S2. Characteristics of Siglec-F expressing neutrophils
Figure S3. Proinflammatory cytokine analysis of injured renal tissue
Figure S4. Cytokine and collagen 1 production by Siglec-F$^+$ neutrophils generated in vivo and in vitro
Figure S5. Evaluation of renal injury and fibrosis by the depletion and adoptive transfer of Siglec-F$^+$ neutrophils
Figure S6. Fibrotic changes of control and tumor tissues from nephrectomy specimen of RCC Patients

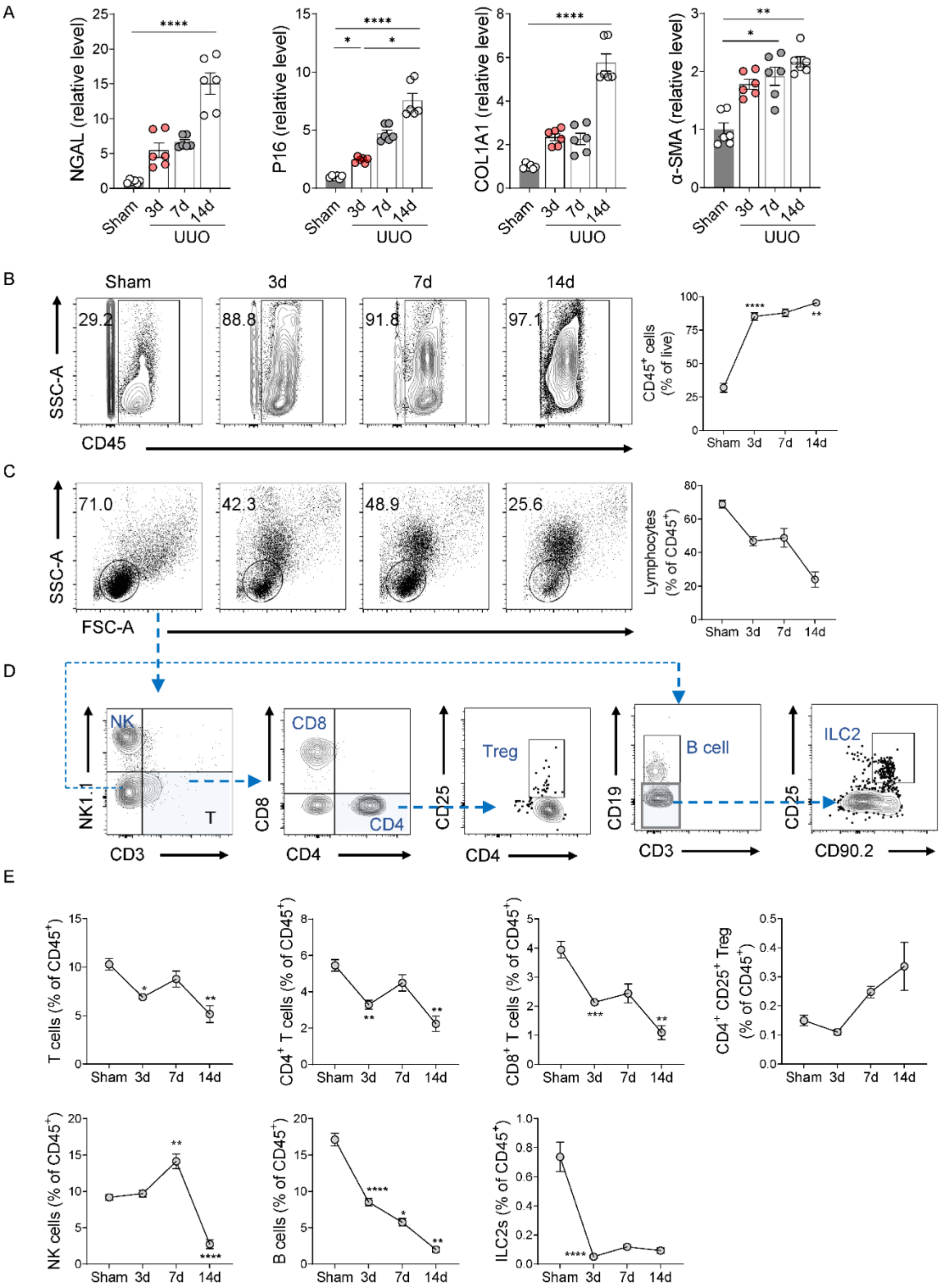

A
NGAL (relative level)
P16 (relative level)
COL1A1 (relative level)
α-SMA (relative level)
Sham 3d 7d 14d
UUO
B
Sham 3d 7d 14d
29.2 88.8 91.8 97.1
SSC-A
CD45
CD45+ cells (% of live)
C
71.0 42.3 48.9 25.6
SSC-A
FSC-A
Lymphocytes (% of CD45+)
D
NK
T
NK1.1
CD3
CD8
CD4
Treg
CD25
B cell
CD19
ILC2
CD90.2
E
T cells (% of CD45+)
CD4+ T cells (% of CD45+)
CD8+ T cells (% of CD45+)
CD4+ CD25+ Treg (% of CD45+)
NK cells (% of CD45+)
B cells (% of CD45+)
ILC2s (% of CD45+)

Figure S1. Inflammation and fibrosis evaluation in UUO-induced kidney injury. UUO induced kidney injury was induced and evaluated on days 0, 3, 7, 14 from the UUO surgery. **(A)** Relative expression of NGAL, P16, COL1A1, and α-SMA evaluated by Western blot. **(B-C)** Frequency of $CD45^+$ leukocytes **(B)** and lymphocytes **(C)** analyzed with flow cytometry. **(D-E)** Immune cell profiling for total T cells, $CD4^+$, $CD8^+$ T cells, regulatory T cells ($CD4^+$ $CD25^+$), NK cells, B cells, and ILC2s ($CD3^-$ $CD19^-$ $NK1.1^-$ $CD90.2^+$ $CD25^+$). All results are shown as mean ± SEM and statistical analysis was performed using one-way ANOVA test. **P*<0.05; ***P*<0.01; ****P*<0.001; *****P*<0.0001; n = 5-7 mice in each group.

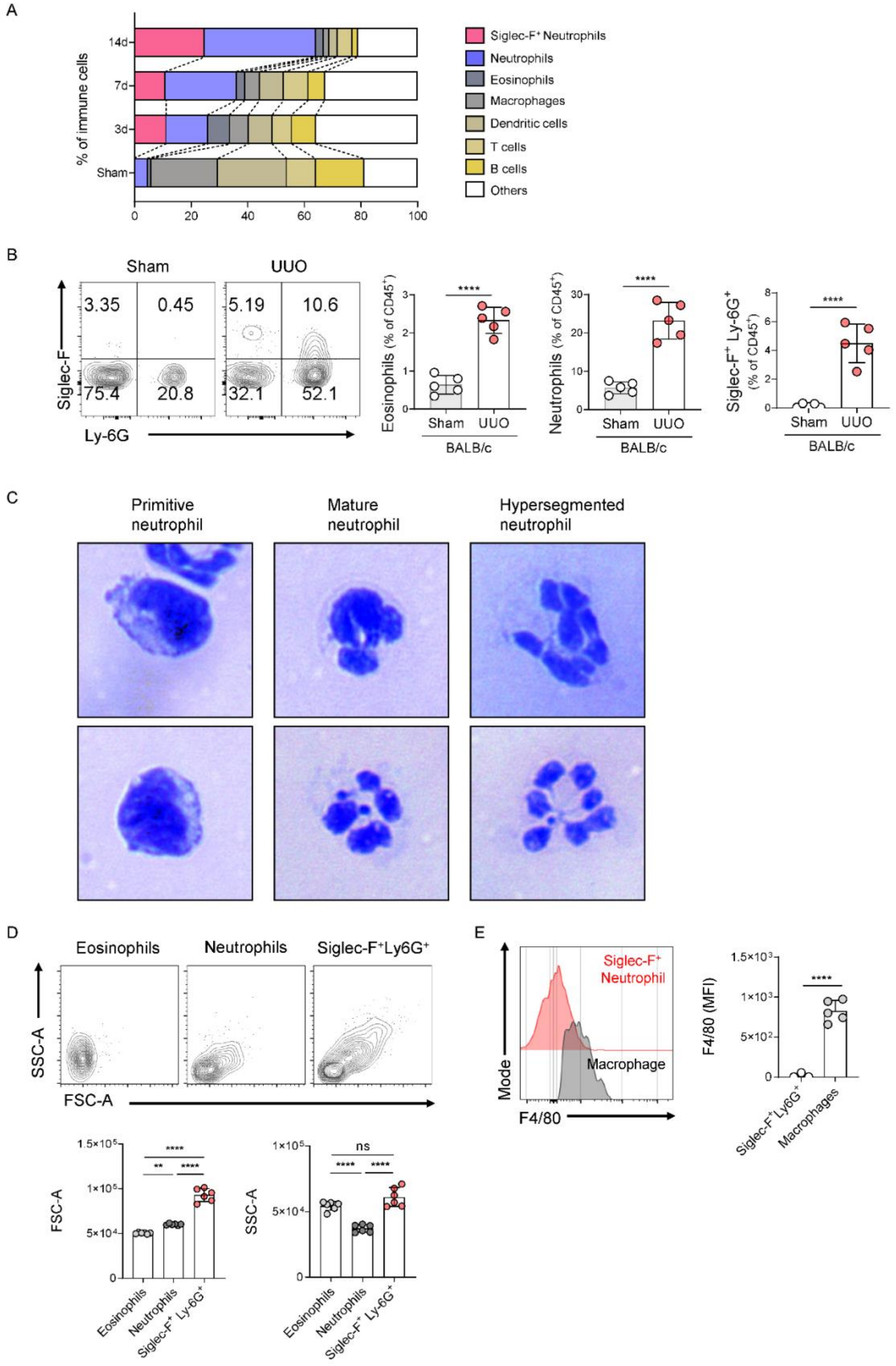
A
% of immune cells
14d
7d
3d
Sham
0
20
40
60
80
100
Siglec-F⁺ Neutrophils
Neutrophils
Eosinophils
Macrophages
Dendritic cells
T cells
B cells
Others
B
Sham
UUO
Siglec-F
Ly-6G
3.35
0.45
75.4
20.8
5.19
10.6
32.1
52.1
Eosinophils (% of CD45⁺)
Neutrophils (% of CD45⁺)
Siglec-F⁺ Ly-6G⁺ (% of CD45⁺)
****
Sham
UUO
BALB/c
C
Primitive neutrophil
Mature neutrophil
Hypersegmented neutrophil
D
Eosinophils
Neutrophils
Siglec-F⁺Ly6G⁺
SSC-A
FSC-A
ns
**
****
Siglec-F⁺ Ly-6G⁺
E
Siglec-F⁺ Neutrophil
Macrophage
Mode
F4/80
F4/80 (MFI)
Siglec-F⁺Ly6G⁺
Macrophages

Figure S2. Characteristics of Siglec-F expressing neutrophils. (A) Kinetic changes of immune cells during UUO-induced renal fibrosis. **(B)** Siglec-F$^+$ Ly-6G$^+$ cells emerged in UUO-induced kidney on BALB/c mice. **(C)** Morphologies of primitive, mature, and hypersegmented neutrophils sorted on day 14. **(D)** Forward scatter (FSC) and side scatter (SSC) comparison between eosinophils, neutrophils, and Siglec-F$^+$ Ly-6G$^+$ cells. **(E)** Comparison of F4/80 expression between Siglec-F$^+$ Ly-6G$^+$ cells and macrophages from day-7 UUO kidney All results are shown as mean ± SEM and statistical analysis was performed using Mann-Whitney test **(B, E)** or one-way ANOVA test **(D)**. *P<0.05; **P<0.01; ***P<0.001; ****P<0.0001; n = 5-7 mice in each group.

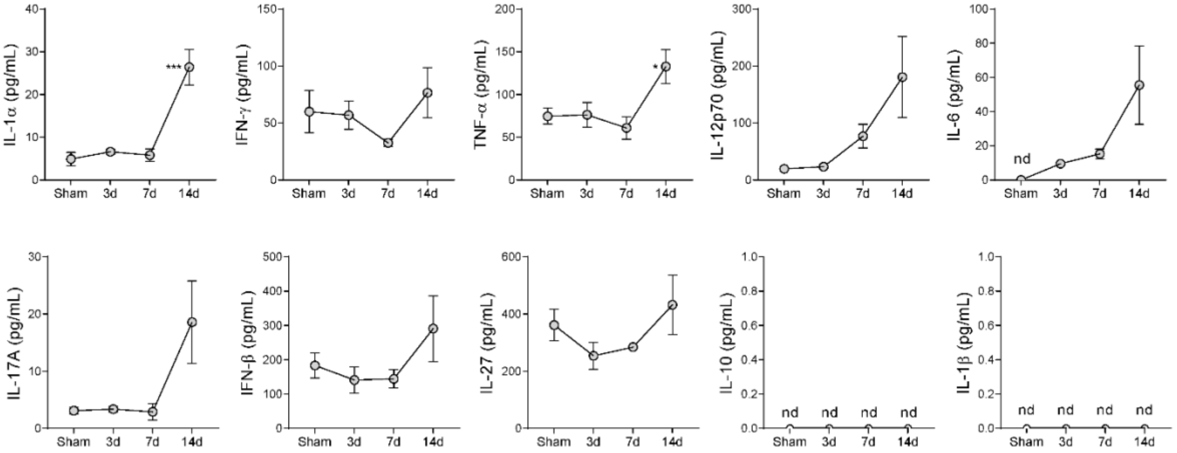

Figure S3. Proinflammatory cytokine analysis of injured renal tissue. Cytokine bead arrays were used to analyze the proinflammatory cytokines increased in the UUO induced renal injury. All results are shown as mean ± SEM and statistical analysis was performed using one-way ANOVA test. **P*<0.05; ****P*<0.001; n = 4-5 mice in each group. ns, not significant.

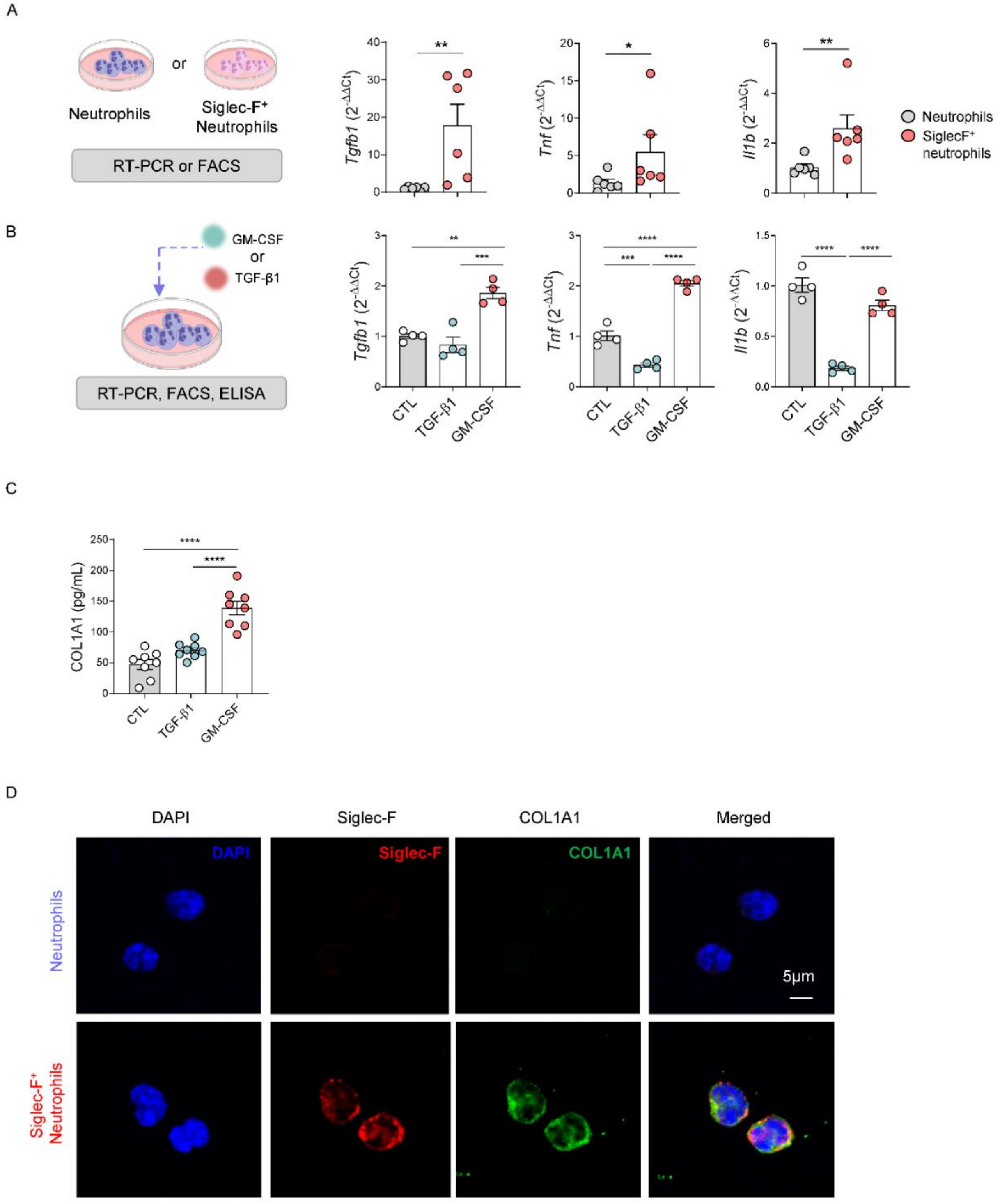
A
or
Neutrophils
Siglec-F+ Neutrophils
RT-PCR or FACS
Tgfb1 (2-ΔΔCt)
Tnf (2-ΔΔCt)
Il1b (2-ΔΔCt)
Neutrophils
SiglecF+ neutrophils
B
GM-CSF
or
TGF-β1
RT-PCR, FACS, ELISA
CTL
TGF-β1
GM-CSF
C
COL1A1 (pg/mL)
D
DAPI
Siglec-F
COL1A1
Merged
Neutrophils
Siglec-F+ Neutrophils
5μm

Figure S4. Cytokine and collagen 1 production by Siglec-F$^+$ neutrophils generated in vivo and in vitro. (A) RT-qPCR analysis of sorted neutrophils and Siglec-F$^+$ neutrophils from UUO-induced kidney (n = 6 in each group). **(B)** RT-qPCR analysis of TGF-β1 or GM-CSF primed neutrophils (n = 4 in each group). **(C-D)** COL1A1 expression of TGF-β1 or GM-CSF primed neutrophils evaluated by ELISA (n = 8 in each group) **(C)** and immunofluorescence **(D)**. All results are shown as mean ± SEM and statistical analysis was performed using Mann-Whitney test (A) or one-way ANOVA test (B-C). *P<0.05; **P<0.01; ***P<0.001; ****P<0.0001.

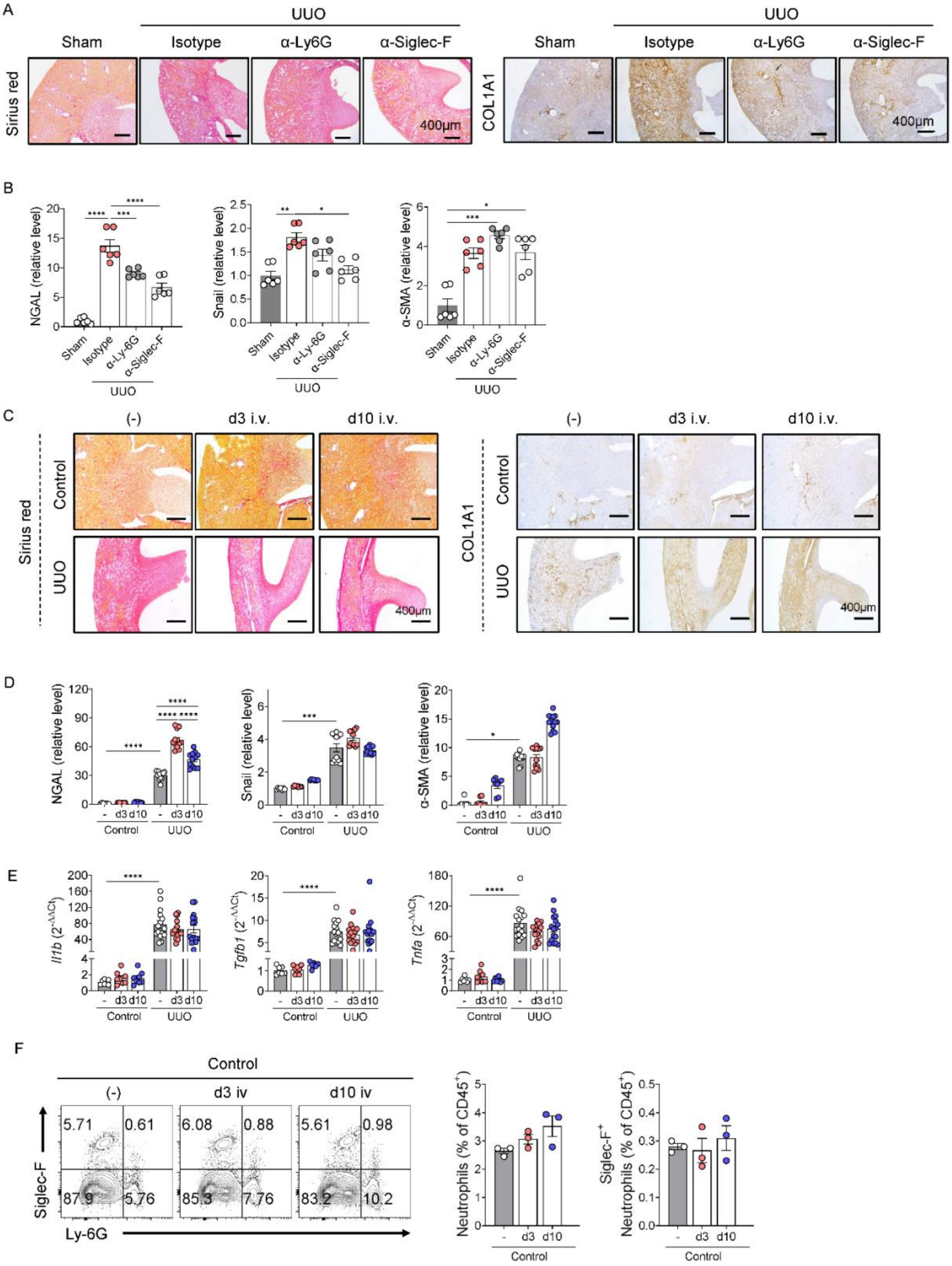
A
UUO
Sham
Isotype
α-Ly6G
α-Siglec-F
Sirius red
400μm
COL1A1
B
NGAL (relative level)
Snail (relative level)
α-SMA (relative level)
Sham
Isotype
α-Ly6G
α-Siglec-F
UUO
C
(-)
d3 i.v.
d10 i.v.
Control
UUO
Sirius red
COL1A1
400μm
D
NGAL (relative level)
Snail (relative level)
α-SMA (relative level)
- d3 d10 - d3 d10
Control UUO
E
Il1b (2^-ΔΔCt)
Tgfb1 (2^-ΔΔCt)
Tnfa (2^-ΔΔCt)
- d3 d10 - d3 d10
Control UUO
F
Control
(-)
d3 iv
d10 iv
5.71 0.61
87.9 5.76
6.08 0.88
85.3 7.76
5.61 0.98
83.2 10.2
Siglec-F
Ly-6G
Neutrophils (% of CD45+)
Siglec-F+ Neutrophils (% of CD45+)
- d3 d10
Control

Figure S5. Evaluation of renal injury and fibrosis by the depletion and adoptive transfer of Siglec-F+ neutrophils. (A-B) Sirius red and COL1A1 staining **(A)**, and NGAL, Snail, and α-SMA expression **(B)** of Siglec-F^+ neutrophil depleted UUO mice. **(C-D)** Sirius red and COL1A1 staining **(C)**, and NGAL, Snail, and α-SMA expression **(D)** of control or UUO mice adoptively transferred with Siglec-F^+ neutrophils. **(E)** Proinflammatory cytokines expressed on renal tissues of Siglec-F^+ neutrophil-transferred control or UUO mouse kidneys. **(F)** Conventional and Siglec-F+ neutrophils in Siglec-F+ neutrophil transferred control mouse kidneys. All results are shown as mean ± SEM and statistical analysis was performed using one-way ANOVA test. **P*<0.05; ***P*<0.01; ****P*<0.001; *****P*<0.0001; n = 6-7 mice in each group for A-B, n=7-16 mice in each group for C-E. and n=3 mice in each group for F.

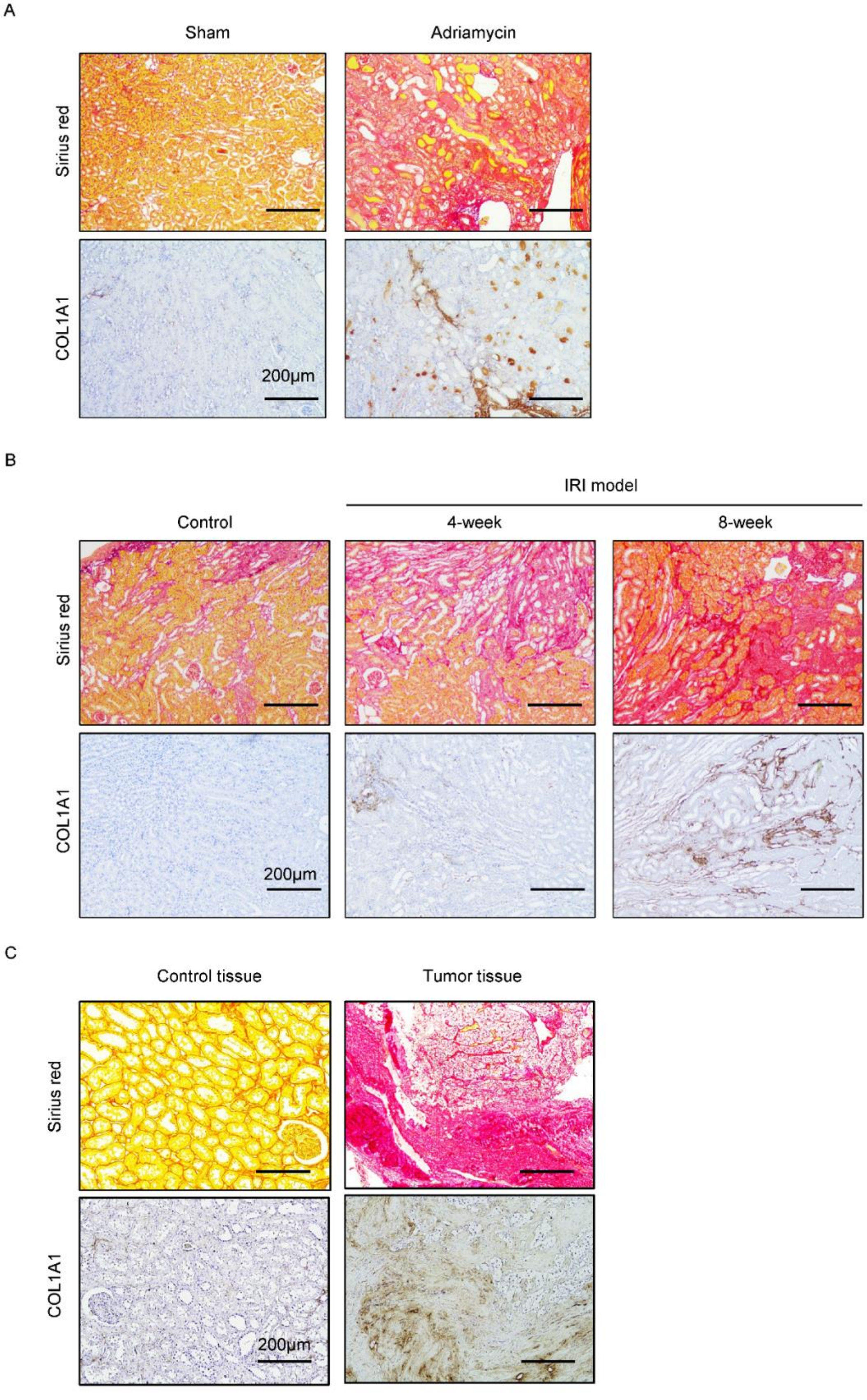
A
Sham
Adriamycin
Sirius red
COL1A1
200µm
B
IRI model
Control
4-week
8-week
Sirius red
COL1A1
200µm
C
Control tissue
Tumor tissue
Sirius red
COL1A1
200µm

Figure S6. Fibrotic changes of other murine CKD models and tumor tissues from nephrectomy specimen of RCC patients. Sirus-red and COL1A1 immunohistochemistry of **(A)** Adriamycin model, **(B)** renal IRI model, and **(C)** tumor tissues from RCC patients.
